# Topotactic anion-exchange in thermoelectric nanostructured layered tin chalcogenides with reduced selenium content†
†Electronic supplementary information (ESI) available. See DOI: 10.1039/c7sc05190e


**DOI:** 10.1039/c7sc05190e

**Published:** 2018-03-23

**Authors:** Guang Han, Srinivas R. Popuri, Heather F. Greer, Ruizhi Zhang, Lourdes Ferre-Llin, Jan-Willem G. Bos, Wuzong Zhou, Michael J. Reece, Douglas J. Paul, Andrew R. Knox, Duncan H. Gregory

**Affiliations:** a WestCHEM , School of Chemistry , University of Glasgow , Glasgow , G12 8QQ , UK . Email: Duncan.Gregory@glasgow.ac.uk; b Institute of Chemical Sciences , Centre for Advanced Energy Storage and Recovery , School of Engineering and Physical Sciences , Heriot-Watt University , Edinburgh , EH14 4AS , UK; c EaStCHEM , School of Chemistry , University of St Andrews , St Andrews , Fife KY16 9ST , UK; d School of Engineering & Materials Science , Queen Mary University of London , London , E1 4NS , UK; e School of Engineering , University of Glasgow , Glasgow , G12 8LT , UK

## Abstract

Anion exchange has been performed with nanoplates of tin sulfide (SnS) *via* “soft chemical” organic-free solution syntheses to yield layered pseudo-ternary tin chalcogenides on a 10 g-scale. SnS undergoes a topotactic transformation to form a series of S-substituted tin selenide (SnSe) nano/micro-plates with tuneable chalcogenide composition. SnS_0.1_Se_0.9_ nanoplates were spark plasma sintered into phase-pure, textured, dense pellets, the *ZT* of which has been significantly enhanced to ≈1.16 from ≈0.74 at 923 K *via* microstructure texturing control. These approaches provide versatile, scalable and low-cost routes to p-type layered tin chalcogenides with controllable composition and competitive thermoelectric performance.

## Abstract 2

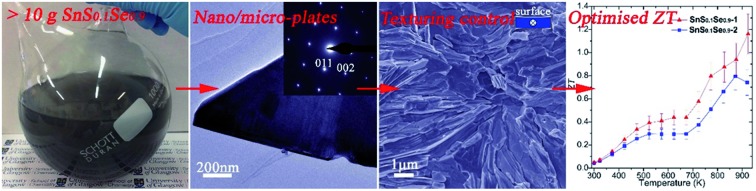
Topotactic solution synthesis yields nanostructured tin chalcogenides, SnS_1–*x*_Se*_x_* with controllable composition; spark plasma sintered SnS_0.1_Se_0.9_ achieves *ZT* ≈ 1.16 at 923 K *via* microstructural texture tuning.

## Introduction

Main group metal chalcogenides (MCs) are excellent candidates for thermoelectrics,^1^–^4^ (opto)electronics^5^ and photovoltaics^6^ due to their outstanding electronic, optical and thermal properties. Bottom-up solution syntheses afford energy-saving means of preparing MC nano/micro-structures with controllable morphology and size. Surface modification using organic surfactants can limit particle growth but such coatings can typically introduce impurities.^7^–^10^ By contrast, synthesis without organic surfactants, solvents or precursors can produce nanostructured MCs with impurity-free surfaces and enhanced electrical performance^11^–^14^ but require careful experiment design with appropriate synthesis parameters and reagents.

Chemical transformations, including ion exchange, topotactic and pseudomorphic reactions, represent a versatile and effective means to produce new materials with control over crystal structure, composition and morphological complexity.^15^ Such transformations can realise prescribed materials that cannot be otherwise prepared.^15^–^17^ Indeed, doped compounds, multi-component composites or hetero-structures can be crafted by regulating the progress of transformations, leading to materials with engineered functional properties.^18^–^21^ When performed in solution, ion exchange can exploit the solubility difference between precursors and products to enable the rapid synthesis of nano/micro-structures (for example, MCs) with predetermined cation and/or anion compositions.^15^,^18^ Combining organic-free synthesis with ion exchange raises the prospect of producing MCs with compositions, crystal structures, morphologies and particle sizes that can be tailored towards delivering high electronic performance.

Thermoelectric materials can be utilised to convert thermal energy directly into electricity and *vice versa*, thus offering opportunities to refrigerate and to harvest electricity from waste heat *via* the Peltier and Seebeck effects, respectively.^22^,^23^ Layered tin chalcogenides (LTCs), including SnSe and SnS, have drawn much attention given a formidable combination of excellent thermoelectric conversion efficiency, relatively low toxicity and the Earth-abundance of their component elements.^1^,^2^,^24^–^26^ Notably, when p-type SnSe can be grown as a single crystal, it has demonstrated record high *ZT* values of 2.6 and 2.3 along the *b* and *c* crystallographic directions, respectively at 923 K.^1^ Polycrystalline SnSe and related doped materials have been prepared in an effort to improve mechanical properties, but *ZT* values cannot yet emulate those in the single crystalline material.^27^ The capacity to synthesise polycrystalline LTCs of premeditated composition to optimise thermoelectric performance is becoming gradually less elusive.^28^–^50^ For example, Ag-doped SnSe,^28^ alkali metal-doped SnSe,^29^–^34^ I-doped SnSe_1–*x*_S_*x*_ (0 ≤ *x* ≤ 1),^35^ and Sn_1–*x*_Pb_*x*_Se^36^ have demonstrated improvements in thermoelectric performance compared to undoped SnSe, pushing *ZT* to 1.2 at 773 K (Na, K co-doped SnSe)^33^ and ∼1.7 at 873 K (phase-separated Sn_1–*x*_Pb_*x*_Se).^36^ Yet, polycrystalline LTCs are primarily fabricated by high-temperature, energy-intensive processes.^27^–^29^,^31^,^32^,^35^ Solution syntheses are an attractive alternative but generally involve using organics (solvents and/or surfactants, for example), can produce small sample yields and have offered little opportunity as yet to exert control over composition.^51^–^60^ For LTCs to be a practicable component of thermoelectric devices, a scalable and cost-effective organic-free synthesis approach to materials with tuneable composition and consistently excellent performance is essential.

In this study, we demonstrate how the combination of two organic-free aqueous solution strategies (anion exchange following direct precipitation) can be utilised to synthesise LTC nano/micro-plates with tuneable chalcogenide composition (*e.g.* >10 g SnS and SnS_0.1_Se_0.9_, respectively; Fig. S1 and S2†). The plates can be sintered into textured, dense pellets with competitive thermoelectric performance while partly replacing selenium with less toxic and more Earth-abundant sulfur.

## Results and discussion

### Characterisation and formation mechanism of anion-exchanged SnS_1–*x*_Se_*x*_

**Fig. 1:**
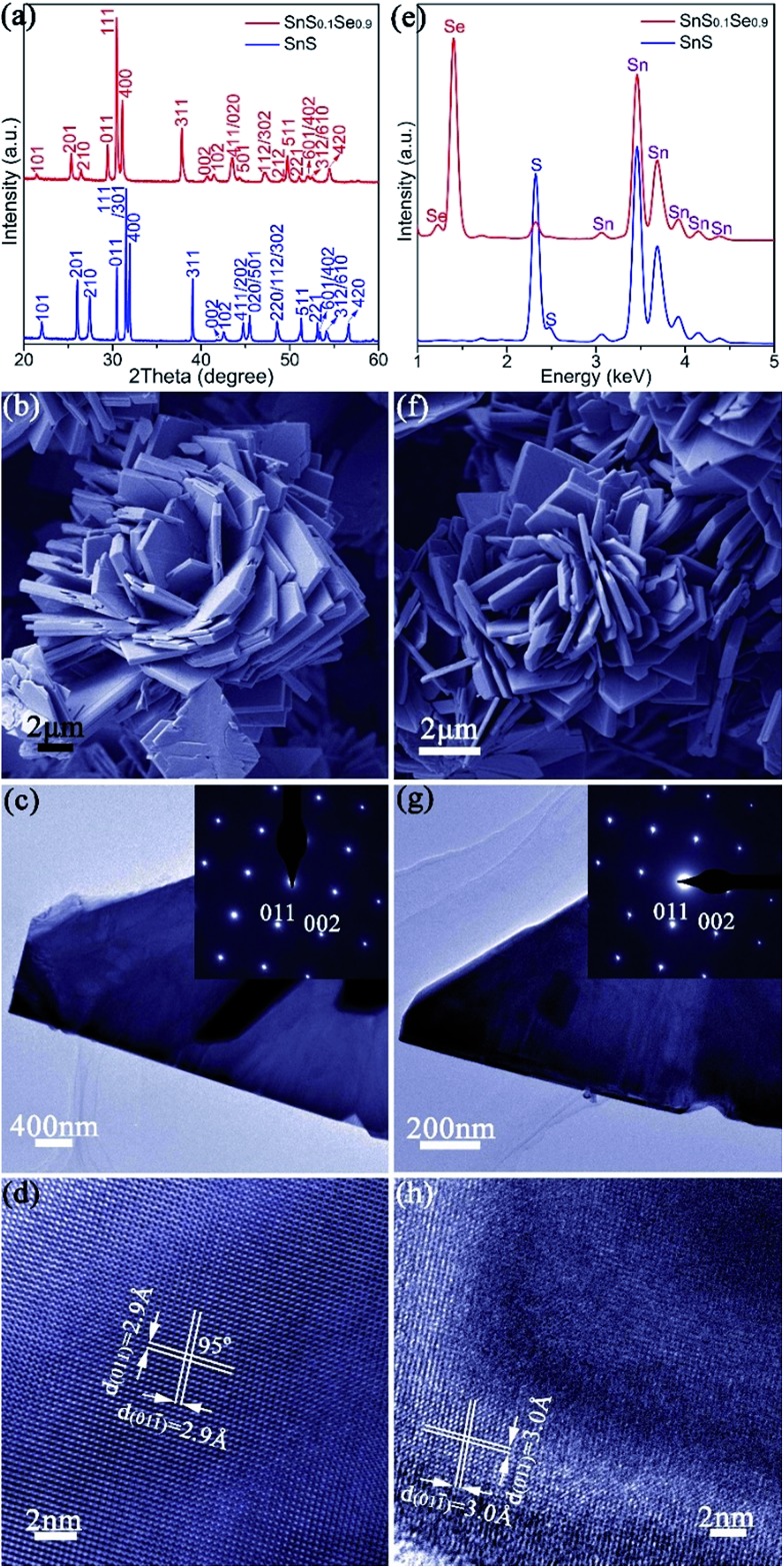
Characterisation of SnS (a–e) and SnS_0.1_Se_0.9_ (a, e–h) nano/micro-plates: (a) PXD data; (b, f) SEM images; (c, g) TEM images of individual plates and corresponding SAED patterns along the [100] zone axis (inset); (d, h) HRTEM images of plates with *d*-spacings indicated, (e) EDS spectra collected from isolated plates.

The synthesis of phase-pure SnS (Fig. 1) involves injection of a Na_2_S aqueous solution into a Na_2_SnO_2_ solution that is subsequently boiled for 2 h (Fig. S1†). Powder X-ray diffraction (PXD) patterns (Fig. 1a) can be indexed exclusively to orthorhombic SnS (ICDD card no. 75-2115).^61^ Rietveld refinement against PXD data (Fig. S3; Tables S1 and S2†) confirms that the SnS product crystallises with orthorhombic space group *Pnma*; *a* = 11.2052(4) Å, *b* = 3.9877(2) Å and *c* = 4.3242(2) Å. Scanning electron microscopy (SEM) images (Fig. 1b and S4a–c†) reveal that the product predominantly forms flower-like nano/micro-structures that are composed of a series of plates, typically with a lateral size of 3–16 μm and a thickness of 80–650 nm. Imaging and electron diffraction of individual nanoplates were performed using transmission electron microscopy (TEM). Selected area electron diffraction (SAED) patterns collected with the incident beam perpendicular to an isolated plate (Fig. 1c) can be indexed to the [100] zone axis of SnS and confirm the single crystalline nature of the nanostructures. High resolution TEM (HRTEM) images (Fig. 1d) demonstrate a set of lattice spacings of 2.9 Å intersecting with an angle of 95(1)°, corresponding to the {011} planes of SnS. Energy dispersive X-ray spectroscopy (EDS) (Fig. 1e and S4d†) confirms Sn : S atomic ratios of 51(1) : 49(1). The organic-free SnS plates can be formed into dense pellets at 500 °C by either hot pressing or spark plasma sintering (SPS), each leading to strong texturing of the (*h*00) planes (Fig. S5 and S6†). The plate morphology, orthorhombic crystal structure and the overall Sn : S ratio are preserved on pressing (Fig. S7†). A pellet of the SnS material has an indirect optical bandgap of *ca.* 1.05 eV (Fig. S8†) and exhibits a negligible weight loss up to *ca.* 600 °C when heated under Ar gas, indicating excellent thermal stability (Fig. S9 and S10†).

**Fig. 2:**
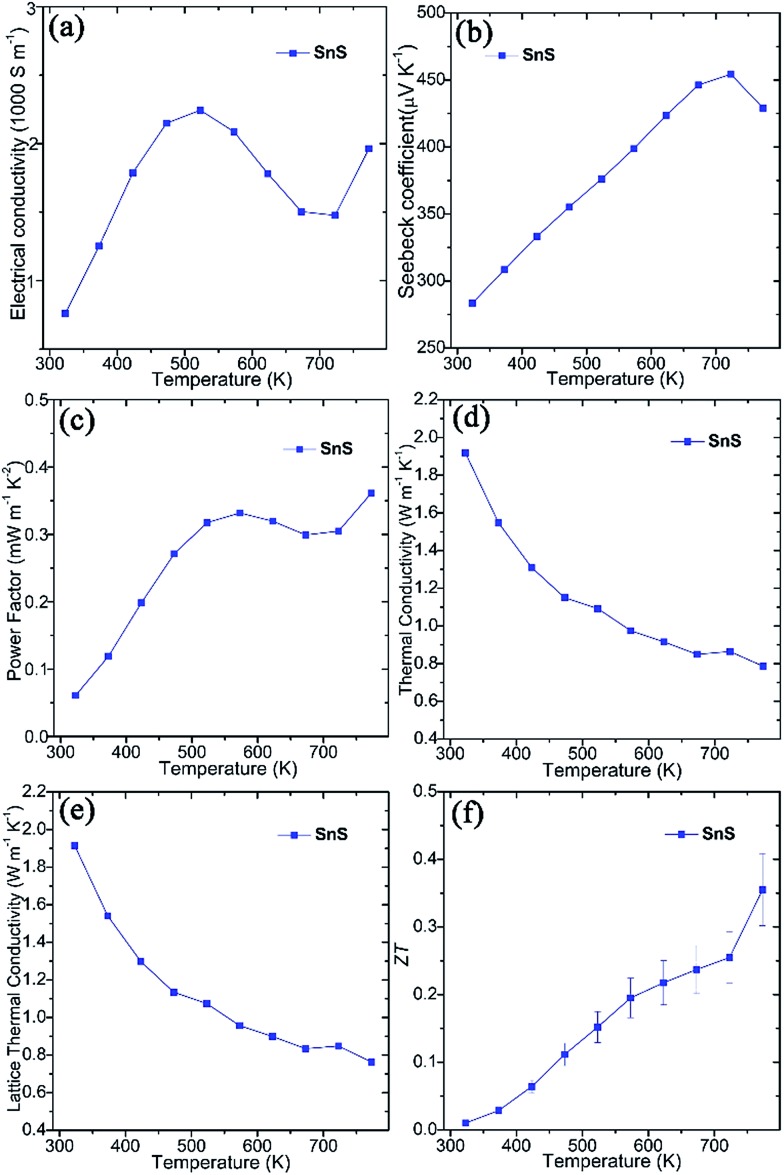
Thermoelectric properties of SnS pellets sintered using SPS, measured perpendicular to the pressing direction: (a) the electrical conductivity (*σ*), (b) the Seebeck coefficient (*S*), (c) the power factor (*S*^2^*σ*), (d) the thermal conductivity (*κ*), (e) the lattice *κ* (*κ*_L_), and (f) *ZT* as a function of temperature.

Thermoelectric measurements were performed on SnS pellets perpendicular to the pressing direction (Fig. 2). The pellet exhibits high electrical conductivity (*σ*), which rises from *ca.* 760 S m^–1^ at 323 K to *ca.* 2250 S m^–1^ at 523 K, gradually decreases to *ca.* 1475 S m^–1^ at 723 K and once again increases to *ca.* 1960 S m^–1^ at 773 K (Fig. 2a). These values are notably much higher than undoped SnS bulk counterparts (*e.g.* ∼5–32 S m^–1^ at 523 K) synthesised by mechanical alloying^25^ and high-temperature synthesis.^26^ Moreover, they also exceed those of pellets consolidated from solvothermally-synthesised SnS nanorods (*e.g.* ∼590 S m^–1^ at 523 K)^62^ and even Ag-doped SnS pellets (*e.g.* ∼530 S m^–1^ at 523 K).^25^ The high *σ* values of SnS pellets can be attributed to the organic-free surfaces of the plates, the orientation of the plates, high crystallinity and the high degree of densification (Fig. S5–S7†). The positive Seebeck coefficient (*S*) values for SnS pellets indicate p-type conducting behaviour (Fig. 2b). The Seebeck coefficient for SnS increases nearly linearly from *ca.* 285 μV K^–1^ at 323 K to *ca.* 455 μV K^–1^ at 723 K before decreasing to *ca.* 430 μV K^–1^ at 773 K. The combination of such high electrical conductivity and Seebeck coefficients leads to power factors (*e.g.* ∼0.33 mW m^–1^ K^–2^ at 573 K and ∼0.36 mW m^–1^ K^–2^ at 773 K) (Fig. 2c) that exceed those of previously reported undoped SnS pellets (*e.g.* ∼0.01–0.10 mW m^–1^ K^–2^ at 573 K).^25^,^26^,^62^ The value of thermal conductivity (*κ*) for SnS reduces from ≈1.918 W m^–1^ K^–1^ at 323 K to ≈0.786 W m^–1^ K^–1^ at 773 K (Fig. 2d), to which the lattice thermal conductivity (*κ*_L_) is the main contributor (Fig. 2e). The *ZT* of a SnS pellet was thus calculated, increasing gradually from *ca.* 0.01 at 323 K to *ca.* 0.36 at 773 K (Fig. 2f). The *ZT* at 773 K is higher than the values reported for undoped SnS (*e.g.* 0.08–0.25 at 773 K) fabricated by various methods,^25^,^26^,^62^ indicating the significant potential of the organic-free method in synthesising high-performing metal chalcogenide thermoelectrics.

To demonstrate the utility of the organic-free strategy in obtaining a range of thermoelectrics with prescribed microstructure and tailored composition, we focused on the synthesis of substituted Sn(S,Se) materials, conscious that thermoelectric properties should improve at higher Se concentration.^26^,^35^ We achieved this *via* an organic-free anion exchange that involved injecting a NaHSe solution into the SnS suspension, followed by boiling for 2 h (Fig. S2†). Samples were prepared with NaHSe : Na_2_SnO_2_ in the appropriate molar ratios with the intention of preparing SnS_1–*x*_Se_*x*_ (0.5 ≤ *x* ≤ 1) (eqn (1) and (2)).

1 Na_2_S + Na_2_SnO_2_ + 2H_2_O → SnS + 4NaOH

2 SnS + *x*NaHSe + *x*NaOH → SnS_1–*x*_Se_*x*_ + *x*Na_2_S + *x*H_2_O

**Fig. 3:**
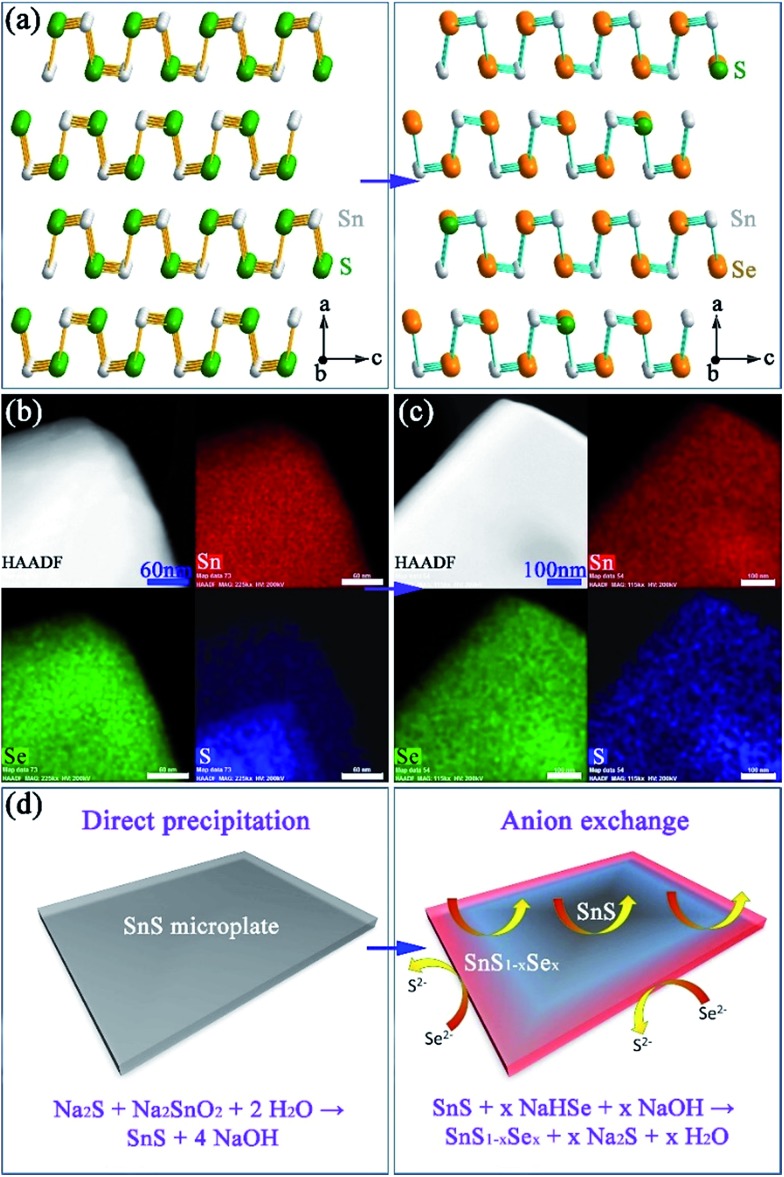
Structure, composition and formation mechanism of Sn(S,Se): (a) structural models of SnS and SnS_0.1_Se_0.9_; elemental mapping of isolated plates after anion exchange for (b) 1 min and (c) 2 h; (d) schematics of the initial precipitation and subsequent exchange processes. The four panels in (b) and (c) show the high angle annular dark field (HAADF) image of a plate and corresponding elemental maps for Sn (red), Se (green) and S (blue) respectively.

The anion exchange is likely driven by the lower solubility product (*K*_sp_) of SnSe in water as compared to that of SnS.^12^,^15^ The PXD patterns of the anion-exchanged products ostensibly resemble that of orthorhombic SnS but with reflections shifted to lower 2*θ* (*e.g.* for *x* = 0.9 in Fig. 1a).^61^ SEM (Fig. 1f and S11a†) reveals that the product nano/micro- “flowers” consist of plates of approximately similar size (2–10 μm) and thickness (50–500 nm) to the original SnS plates. EDS spectra collected from both individual plates and clusters of plates in the 1 : 1, S : Se material consistently gave Sn : Se : S atomic ratios of 50(1) : 45(1) : 5(1) (Fig. 1e and S11b†). Rietveld refinement (Fig. S12; Tables S3 and S4†) confirms that the product crystallises with smaller cell parameters than SnSe (orthorhombic, *Pnma*; *a* = 11.4919(4) Å, *b* = 4.1507(2) Å, *c* = 4.4334(2) Å) with an anion site occupancy of 0.91(1) : 0.09(1) Se : S, corresponding to a composition close to SnS_0.1_Se_0.9_ and includes 1.4(1) wt% of SnS as a secondary phase. SAED patterns along the [100] zone axis (Fig. 1g) combined with HRTEM images (Fig. 1h) (which show lattice spacings of 3.0 Å with an intersection angle of 94(1)°, corresponding to the SnS_0.1_Se_0.9_ {011} plane spacings) identify preferred orientation along the 100 direction. Hence both the morphology and crystal structure before and after anion exchange are essentially unaltered and the process is topotactic (Fig. 3a).^18^

**Fig. 4:**
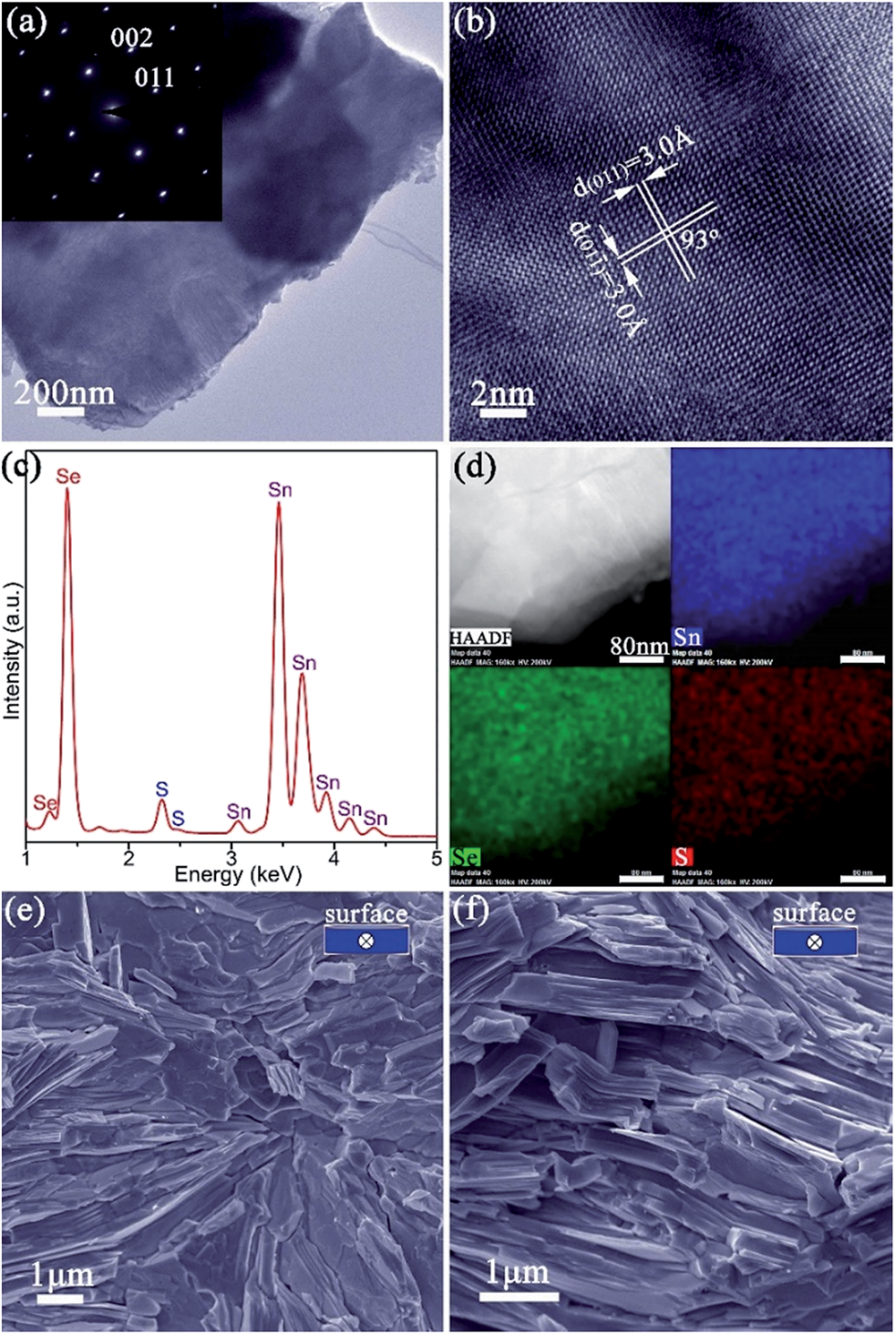
Characterisation of SnS_0.1_Se_0.9_ pellets: (a) TEM image of a SnS_0.1_Se_0.9_ plate peeled from a pellet and its corresponding SAED pattern along the [100] zone axis; (b) HRTEM image of the plate shown in (a) with *d*-spacings indicated; (c) EDS spectrum from the plate in (a); (d) elemental mapping of the same plate; (e, f) SEM images of the fractured cross sections of SnS_0.1_Se_0.9_-1 and SnS_0.1_Se_0.9_-2 with the viewing direction (parallel to pellet surface) indicated in the inset. The four panels in (d) are the HAADF image and corresponding elemental maps for Sn (blue), Se (green) and S (red) respectively.

In an effort to understand the formation mechanism of SnS_0.1_Se_0.9_, we investigated the outcome of an anion exchange reaction after only 1 min of boiling (using a Se : Sn molar ratio of 1). PXD shows that the product consists of both SnS_1–*x*_Se_*x*_ (*x* ≈ 0.9) and SnS (Fig. S13a†). SEM (Fig. S13b and c†) reveals clusters of nano/micro-plates, indicating again the retention of the SnS morphology. EDS (Fig. S13d†) gives an overall Sn : Se : S atomic ratio of 50(1) : 33(1) : 17(1) across the clusters, implying incomplete anion exchange. EDS element mapping (Fig. 3b) of an isolated plate from the 1 min synthesis reveals an uneven distribution of Se and S where the plate edges are much richer in Se than S (Se : S ratio of 49 : 1; Fig. S14†). Conversely the inner sections contain more S than Se (Se : S ratio of 23 : 27; Fig. S14†). By comparison, systematic elemental mapping of the product boiled for 2 h illustrates that *ca.* 50% of plates show an almost uniform distribution of Se and S (Fig. 3c), while the remainder demonstrate an uneven anion distribution. With Se : S ratios varying from 48(1) : 2(1) to 42(1) : 8(1) (Fig. S15†) the plates in the 2 h anion exchange sample are clearly much richer in Se than the 1 min product. Given the above observations, one can propose a formation process for the SnS_1–*x*_Se_*x*_ plates (Fig. 3d). The anion exchange should initiate at the edges and faces of plates. The periphery of a plate can access Se^2–^ from both the edges and faces and therefore should be richer in Se than the centre. For thicker plates the contribution from anion exchange at the edges should become more pronounced. It is useful to note at this point that both the plate morphology and orthorhombic crystal structure are maintained after the subsequent high temperature sintering (*e.g.* at 500 °C) by either hot pressing or spark plasma sintering (SPS). Moreover, the overall Se : S ratio remains constant and the distribution of S and Se becomes more even across each plate (Fig. 4a–d).

The NaHSe concentration is a crucial synthesis parameter for the synthesis of SnS_1–*x*_Se_*x*_ nano/micro-plates with tuneable S concentration. When the NaHSe : Na_2_SnO_2_ molar ratio is increased from 1 through 1.15 to 1.5, it is possible to obtain almost single-phase orthorhombic SnS_1–*x*_Se_*x*_ with very high Se concentrations (Fig. S16†). The Se site occupancy obtained from Rietveld refinement increases from 0.91(1) through 0.92(2) to 0.94(2) (Table S5†) (corresponding to *x* = 0.91, *x* = 0.92 and *x* = 0.94 respectively). On decreasing the Se : Sn molar ratio (and *x*) from 0.9 through 0.8 to 0.5, the SnS_1–*x*_Se_*x*_ products accordingly contain more sulphide together with rising phase fractions of SnS (Fig. S16; Table S6†). The lattice parameters and unit cell volumes of these products increase with increasing Se concentration (*x*) which follows Vegard's law (Fig. S17†). The products synthesised at various NaHSe concentrations universally take the form of nano/micro-flowers consisting of clusters of nano/micro-plates, reinforcing the premise that the anion exchange is (pseudomorphic and) topotactic (Fig. S18 and S19†). As the NaHSe : Na_2_SnO_2_ molar ratio is increased, EDS spectra show that the Se/(Se + S) ratio increases from ∼0.47, through ∼0.77, ∼0.85, ∼0.89, ∼0.92 to ∼0.93 (Fig. S18–S20†), which is consistent with the Rietveld refinement results.

### Thermoelectric performance of anion-exchanged SnS_1–*x*_Se_*x*_

The ability to prepare >10 g SnS_1–*x*_Se_*x*_ plates, with control over both structure and composition, facilitates the fabrication of textured, dense pellets. Of the various SnS_1–*x*_Se_*x*_ materials synthesised, SnS_0.1_Se_0.9_ was selected for thermoelectric measurements given previously documented electrical and thermal properties of the equivalent bulk material of the same composition.^35^ Pellets with *ca.* 98% of the SnS_0.1_Se_0.9_ theoretical density were consolidated from 2 h anion-exchanged SnS_0.1_Se_0.9_ plates *via* one-step (denoted SnS_0.1_Se_0.9_-1) and two-step (denoted SnS_0.1_Se_0.9_-2) SPS processes. The two-step SPS process can give rise to the superplastic flow of unconstrained samples in the transverse direction (*i.e.* perpendicular to the pressing direction), in turn producing textured samples.^63^ The orthorhombic structure of the materials persists after sintering and both pellets exhibit strong orientation in the (*h*00) plane (Fig. S21†). SnS_0.1_Se_0.9_-2 exhibits much stronger texturing than SnS_0.1_Se_0.9_-1 as evidenced by the higher relative diffraction intensities of the (*h*00) reflections. In fact, the orientation degree (*F*) for (*h*00), estimated by the Lotgering method^64^ and based on the PXD data (Fig. S21a†), is *ca.* 0.25 and *ca.* 0.46, for SnS_0.1_Se_0.9_-1 and SnS_0.1_Se_0.9_-2, respectively, demonstrating the greater texturing in the latter sample. SEM images of the cleavage surfaces (Fig. S22a–d†) and fractured cross-sections (Fig. 4e, f, S22e and f†) reveal that SnS_0.1_Se_0.9_-1 and SnS_0.1_Se_0.9_-2 consist of densely packed, stacked plates. In SnS_0.1_Se_0.9_-2 the plates are aligned almost parallel to the pellet surface (perpendicular to the pressing direction), while those in SnS_0.1_Se_0.9_-1 are more directionally disordered, which is consistent with PXD results. EDS (Fig. S22g and h†) reveals that the pellets conserve their composition post-sintering (Sn : Se : S = 50(1) : 45(1) : 5(1)). Both pellets have an indirect optical bandgap of *ca.* 0.86 eV (Fig. S23†) and exhibit a negligible weight loss (<0.5 wt%) below 700 °C when heated under Ar gas (Fig. S24 and S25†).

**Fig. 5:**
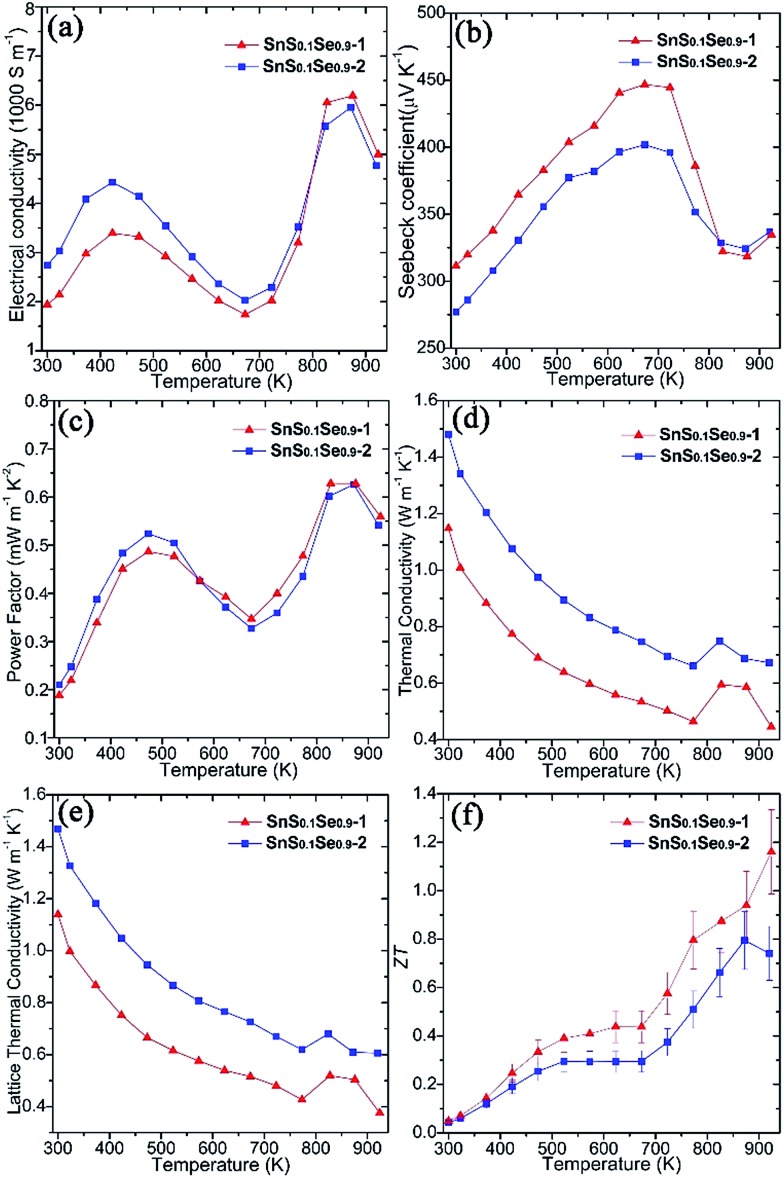
Thermoelectric properties of SnS_0.1_Se_0.9_ pellets SnS_0.1_Se_0.9_-1 and SnS_0.1_Se_0.9_-2 measured perpendicular to the pressing direction: (a) the electrical conductivity (*σ*), (b) the Seebeck coefficient (*S*), (c) the power factor (*S*^2^*σ*), (d) the thermal conductivity (*κ*), (e) the lattice *κ* (*κ*_L_), and (f) *ZT* as a function of temperature.

Thermoelectric measurements were performed on both SnS_0.1_Se_0.9_ pellets perpendicular to the pressing direction (Fig. 5). The electrical conductivity of SnS_0.1_Se_0.9_-1 (Fig. 5a) increases from *ca.* 1940 S m^–1^ at 300 K to *ca.* 3400 S m^–1^ at 423 K, gradually decreases to *ca.* 1740 S m^–1^ at 673 K and reaches a maximum of *ca.* 6200 S m^–1^ at 873 K. The value of *σ* subsequently subsides to *ca.* 5000 S m^–1^ at 923 K. This behaviour has been previously observed in SnSe polycrystalline materials. It has been suggested that the reduction in *σ* over the mid-temperature range followed by a subsequent increase could be related to a reduction in carrier mobility and a thermal excitation of carriers, respectively.^55^,^56^ SnS_0.1_Se_0.9_-2 exhibits very similar behaviour to SnS_0.1_Se_0.9_-1, but demonstrates higher electrical conductivity below *ca.* 823 K (and more notably at lower *T*). As with SnS samples, SnS_0.1_Se_0.9_-1 and SnS_0.1_Se_0.9_-2 demonstrate a combination of clean particle surfaces, pronounced plate orientation, high crystallinity and high density. Coupled with the reduced bandgap engendered by Se doping, the high *σ* values observed can be plausibly rationalised. That SnS_0.1_Se_0.9_-2 exhibits higher conductivity is likely due to the enhanced (*h*00) texturing induced by the two-step SPS process. Electrical conductivity within the Sn–Se layers (in the *bc* plane) of SnSe is expected to be much higher than that perpendicular to the layers (along *a*).^1^ Indeed, the room temperature Hall carrier mobility of SnS_0.1_Se_0.9_-2 (evidenced as more strongly textured) is approximately 20.6% higher than that of SnS_0.1_Se_0.9_-1 (Table 1).

**Table 1:** A summary of electrical properties (*σ*_300 K_ and *S*_300 K_), room temperature Hall carrier concentration (*n*_H_) and mobility (*μ*_H_) of the pellets

Pellet	*σ*_300 K_ [S m^–1^]	*S*_300 K_ [μV K^–1^]	*n*_H_ [10^17^ cm^–3^]	*μ*_H_ [cm^2^ V^–1^ s^–1^]
SnS_0.1_Se_0.9_-1	1942	312	22.5	53.9
SnS_0.1_Se_0.9_-2	2739	277	26.3	65.0

Both pellets exhibit p-type conducting behaviour, demonstrated by the positive Seebeck coefficient (Fig. 5b). *S* for SnS_0.1_Se_0.9_-1 increases almost linearly from *ca.* 310 μV K^–1^ at 300 K reaching a maximum at *ca.* 445 μV K^–1^ at 673 K. By 873 K, *S* drops to *ca.* 320 μV K^–1^ before a slight upturn to *ca.* 335 μV K^–1^ at 923 K. Below *ca.* 823 K, the Seebeck coefficient for SnS_0.1_Se_0.9_-2 is consistently slightly lower than that for SnS_0.1_Se_0.9_-1 but nevertheless follows the same trend with temperature. The decrease in *S* above 673 K could be related to the thermal excitation of minority carriers.^55^,^56^

The combination of large *σ* values coupled with high values of *S* results in exceptional power factors (*S*^2^*σ*) for SnS_0.1_Se_0.9_-1 and SnS_0.1_Se_0.9_-2 (Fig. 5c) which both show local maxima (of *ca.* 0.50 mW m^–1^ K^–2^) at 473 K and (of *ca.* 0.63 mW m^–1^ K^–2^) at 873 K. The *S*^2^*σ vs. T* behaviour hence broadly follows the temperature variation of *σ*, although the power factor for SnS_0.1_Se_0.9_-2 is marginally inferior to SnS_0.1_Se_0.9_-1 above 573 K. Interestingly SnS_0.1_Se_0.9_ and SnS show very similar trends in the variation of *σ*, *S* and *S*^2^*σ* with temperature (Fig. S26†), indicative of the broadly similar crystalline and electronic structures. Ultimately, SnS_0.1_Se_0.9_ reaches a higher value of *σ* (Fig. S26a†), probably primarily due to the reduction in the bandgap as Se replaces S.

Both SnS_0.1_Se_0.9_-1 and SnS_0.1_Se_0.9_-2 have higher power factors than SnSe pellets consolidated from surfactant-free nanoplates (increasing from *ca.* 0.05 mW m^–1^ K^–1^ at 300 K to *ca.* 0.40 mW m^–1^ K^–1^ at 550 K; Fig. S26c†),^11^ but perhaps particularly remarkable is that *S*^2^*σ* for SnS_0.1_Se_0.9_-1 at 773 K exceeds those of other SnS_1–*x*_Se_*x*_ (*x* < 1) materials (*e.g. S*^2^*σ* values at 773 K of ≈0.24 mW m^–1^ K^–2^ for p-type SnS_0.2_Se_0.8_ (ref. 26) and ≈0.40 mW m^–1^ K^–2^ for n-type SnS_0.1_Se_0.87_I_0.03_ (ref. 35)), and matches or surpasses those for the best examples of doped polycrystalline SnSe (*e.g.* 0.39–0.48 mW m^–1^ K^–2^ for p-type Na-doped SnSe at 773 K).^29^,^31^,^32^ Such materials require high-temperature methods to produce, so it is clearly possible to replace these synthesis methods by more efficient, energy-saving alternatives without sacrificing performance.

Microstructural texturing also influences the thermal conductivity (*κ*) of the pellets (Fig. 5d). The value of *κ* for SnS_0.1_Se_0.9_-1 is low, decreasing from ≈1.148 W m^–1^ K^–1^ at 300 K to ≈0.464 W m^–1^ K^–1^ at 773 K. The values for SnS_0.1_Se_0.9_-2 at the equivalent temperatures are approximately 29.0% and 42.4% larger respectively. The increase in *κ* at 823 K is most likely related to the second order displacive phase transformation of SnSe (from orthorhombic *Pnma* to orthorhombic *Cmcm*).^65^ As with the electrical conductivity, the thermal conductivity is anisotropic in SnS_1–*x*_Se_*x*_ and so the difference in *κ* can be attributed to the different degrees of texturing in SnS_0.1_Se_0.9_-1 and SnS_0.1_Se_0.9_-2. The thermal conductivity along the *a*-axis (perpendicular to the Sn–Se planes) is much lower than that along *b* or *c*.^1^ The main contribution to *κ* in the pellets is the lattice thermal conductivity (*e.g. κ*_L_ ≈ 0.427 W m^–1^ K^–1^ for SnS_0.1_Se_0.9_-1 and *κ*_L_ ≈ 0.620 W m^–1^ K^–1^ for SnS_0.1_Se_0.9_-2 at 773 K) (Fig. 5e) and the magnitude of *κ* and *κ*_L_ demonstrates the degree to which texturing can influence the thermal properties of polycrystalline materials such as SnSe. It should be noted that *κ*_L_ is still higher than the theoretical minimum for SnSe (*κ*_L_ ≈ 0.26 W m^–1^ K^–1^ at 770 K),^35^ so one might expect that by further texture control and/or by reducing the plate dimensions, *κ*_L_ could be driven further towards this theoretical minimum. It is also interesting to note that SnS (containing a lighter chalcogen), has a higher *κ*_L_ than SnS_0.1_Se_0.9_ across the whole measurement temperature range (Fig. S26e†).

*ZT* could be calculated for the pellets using the above electrical and thermal transport data (Fig. 5f). The *ZT* of SnS_0.1_Se_0.9_-1 is the higher across the whole *T* range, increasing from *ca.* 0.05 at 300 K to *ca.* 1.16 at 923 K; the *ZT* of SnS_0.1_Se_0.9_-2 reaches a maximum of *ca.* 0.80 at 873 K. The *ZT* of SnS_0.1_Se_0.9_-1 at 923 K is comparable to SnSe bulk materials with carbon inclusions measured at 903 K.^37^ Considering previous reports of the thermoelectric performance of SnSe up to 800 K, it is useful to compare the value of *ZT* close to this elevated temperature. The value for SnS_0.1_Se_0.9_-1 at 773 K is higher than that of sulfur-free p-type polycrystalline SnSe (*ZT* ≈ 0.39–0.66)^27^,^66^ and comparable to various metal-doped, p-type polycrystalline SnSe (*ZT* ≈ 0.5–1.2)^26^,^28^–^32^,^36^ (where all measurements were made perpendicular to the pressing direction at approximately 773 K; *e.g.* ≈0.6 for Ag-doped SnSe at 750 K,^28^ ≈0.8 for Na-doped SnSe at 800 K,^31^ ≈1.1 for K-doped SnSe at 773 K).^30^ Importantly, this indicates that tin chalcogenides can retain high thermoelectric performance when some of the more toxic Se is removed and replaced by less toxic S *via* anion exchange. Further systematic study of the thermoelectric performance of SnS_1–*x*_Se_*x*_ as a function of *x* should reveal the optimum materials and processing parameters in the system. Moreover, it may be possible to offset loss of performance with decreasing *x* by co-doping with appropriate low toxicity, Earth-abundant metals. Although homogeneity of the materials is realised in SPS treated pellets, we note that the extent of anion exchange during synthesis will be governed primarily by reaction kinetics. Both the kinetics and thermodynamics (*K*_sp_) could be modified by replacing water with another solvent, but given the economic and environmental advantages in using aqueous chemistry, modifying reaction temperature (and/or autogenous pressure, hydrothermally) would be the obvious parameter(s) to investigate towards achieving complete topotactic conversion (Fig. 3d). There is thus considerable scope for polycrystalline SnSe materials to achieve (average) *ZT* values comparable to analogous single crystals. Given the materials design options to reduce *κ* (and notably *κ*_L_) further as described above (and for example, *via* precipitates^30^) and to increase carrier concentration (to the magnitude of 10^19^ cm^–3^) and electrical conductivity, *σ* (*e.g.* through alkali ion doping^2^,^24^,^29^,^31^,^33^), it should be possible to propel *ZT* to still higher reaches in tin chalcogenides without sacrificing green chemistry principles.

## Conclusions

In summary, organic-free, scalable, low-cost solution approaches have been developed to produce nanostructured layered tin chalcogenides with tuneable chalcogenide composition. This precipitation-anion exchange protocol yielded SnS_0.1_Se_0.9_ plates, which were consolidated into p-type textured pellets, achieving *ZT* ≈ 1.16 at 923 K through tuning the microstructural texturing. The precipitation-anion exchange route provides a versatile means to modify the microstructure and composition of layered tin chalcogenides directly from solution. Both variables have profound effects on the transport properties and thermoelectric performance of the resulting materials. The method should be extendable to the synthesis of a host of other p-block metal chalcogenides and provide scope for a wide-ranging strategy invoking nanostructuring and doping as fundamental parameters.

## Experimental

Full experimental details are provided in the ESI.†

### Materials synthesis

150 mmol NaOH and 10 mmol SnCl_2_·2H_2_O were added into 50 ml deionised water (DIW) to yield a transparent Na_2_SnO_2_ solution that was then heated to boil. 40 ml of Na_2_S_(aq)_ (0.5 mol L^–1^) was promptly injected into the boiling solution, which was then further boiled for 2 h. The reaction was terminated at this point for the synthesis of SnS, while for the synthesis of SnS_1–*x*_Se_*x*_ materials such as SnS_0.1_Se_0.9_, used as an example here, 40 ml of freshly prepared NaHSe_(aq)_ (0.25 mol L^–1^) was promptly injected into the SnS suspension that was boiled for another 2 h and then cooled to room temperature. Heating and cooling of the solution were performed under Ar_(g)_ on a Schlenk line. The products were washed with DIW and ethanol and dried at 50 °C for 12 h. Scaled-up syntheses of SnS and SnS_0.1_Se_0.9_ were performed with eight-fold and six-fold precursor concentrations, respectively. SnS pellets (density of 5.17 g cm^–3^) were sintered in a graphite die (diameter: 15 mm) under vacuum by SPS (FCT HP D 25, FCT System GmbH; uniaxial pressure of 60 MPa; 500 °C; 5 min). SnS_0.1_Se_0.9_ pellets (density of 5.95 g cm^–3^) were sintered in a graphite die under vacuum by SPS in either a 1-step (uniaxial pressure of 60 MPa; 500 °C; 5 min; die diameter: 15 mm) or 2-step (1^st^ step: 50 MPa; 450 °C; 5 min; die diameter: 15 mm. 2^nd^ step: 50 MPa; 500 °C; 5 min; die diameter: 20 mm) process.^63^

### Materials characterisation and testing

PXD data were collected with a PANalytical X'pert Pro MPD diffractometer in Bragg–Brentano geometry (Cu Kα_1_ radiation, *λ* = 1.5406 Å). Rietveld refinement was performed against PXD data using the GSAS and EXPGUI software packages.^67^,^68^ Imaging and elemental analysis were conducted by SEM (Carl Zeiss Sigma, at 5 and 20 kV respectively) equipped with EDS (Oxford Instruments X-Max 80). Further imaging, elemental analysis and SAED were performed by TEM (FEI Titan Themis 200 equipped with Super-X windowless EDS detector, operated at 200 kV). Thermogravimetric-differential thermal analysis (TG-DTA) of the samples was performed using a Netzsch STA 409 thermal analyser under flowing Ar. Optical bandgaps were measured by DR-UV-Vis spectroscopy (Shimadzu, UV-2600). All thermoelectric measurements were made perpendicular to the pressing direction. Following prior determination of thermal stability and pre-heating treatments if necessary, the Seebeck coefficient (*S*) and electrical conductivity (*σ*) of pellets were measured simultaneously using a Linseis LSR-3 instrument from 300–923 K. The thermal conductivity, *κ* of the pellets was calculated by *κ* = *DC*_p_*ρ*, where *D*, *C*_p_ and *ρ* are the thermal diffusivity coefficient, specific heat capacity and density, respectively. *D* of the SnS_0.1_Se_0.9_ pellets was measured using a Netzsch LFA 457 from 300–923 K (Fig. S27a†); *C*_p_ of SnS_0.1_Se_0.9_ (Fig. S27b†) was calculated from the weighted average^26^ of reported *C*_p_ of SnSe^1^ and SnS.^25^*ρ* of the pellets was measured by the Archimedes method. Electronic thermal conductivity (*κ*_e_) was estimated by the Wiedemann–Franz law (*κ*_e_ = *LσT*, where *L* is the Lorentz number; *L* of 1.5 × 10^–8^ V^2^ K^–2^ was applied^1^), and lattice thermal conductivity (*κ*_L_) was calculated by subtracting *κ*_e_ from *κ*. *ZT* was calculated *via ZT* = *S*^2^*σT*/*κ*. Hall measurements were performed on a nanometrics HL5500 Hall system using a van der Pauw configuration.

## Conflicts of interest

There are no conflicts to declare.

## Supplementary Material

Supplementary information
