# Supplementary material for: Topotactic anion-exchange in thermoelectric nanostructured layered tin chalcogenides with reduced selenium content
Source: Chem Sci. 2018 Mar 23;9(15):3828–36. doi: 10.1039/c7sc05190e (PMC5939836; doi:10.1039/c7sc05190e)
Supplement: Supplementary file 1 [file SC-009-C7SC05190E-s001.pdf]

Electronic Supplementary Material (ESI) for Chemical Science.
This journal is © The Royal Society of Chemistry 2018

Topotactic Anion-exchange in Thermoelectric Nanostructured Layered Tin Chalcogenides with Reduced Selenium Content; *Supporting Information.*

Guang Han,[a] Srinivas R. Popuri,[b] Heather F. Greer,[c] Ruizhi Zhang,[d] Lourdes F. Llin,[e] Jan-Willem G. Bos,[b] Wuzong Zhou,[c] Michael J. Reece,[d] Douglas J. Paul,[e] Andew R. Knox,[e] Duncan H. Gregory[a*]

[a]WestCHEM, School of Chemistry, University of Glasgow, Glasgow, G12 8QQ, UK

[b]Institute of Chemical Sciences and Centre for Advanced Energy Storage and Recovery, School of Engineering and Physical Sciences, Heriot-Watt University, Edinburgh, EH14 4AS, UK

[c]EaStCHEM, School of Chemistry, University of St Andrews, St Andrews, Fife KY16 9ST, UK

[d]School of Engineering & Materials Science, Queen Mary University of London, London, E1 4NS, UK

[e]School of Engineering, University of Glasgow, Glasgow, G12 8QQ, UK

*Corresponding author: Email: Duncan.Gregory@glasgow.ac.uk

Experimental details

Materials Synthesis. In a typical synthesis of SnS nano/micro-plates, 150 mmol NaOH (Sigma, 99.99%) and 10 mmol $SnCl_2 \cdot 2H_2O$ (Sigma, 99.99%) were added into 50 ml deionised water (DIW) within a two-neck round-bottom flask to yield a transparent Na_2SnO_2 solution. In parallel, 20 mmol Na_2S (Alfa, >96.2%) was added into 40 ml DIW within a single-neck round-bottom flask, to prepare a light yellow solution. After the Na_2SnO_2 solution was heated to its boiling temperature under reflux using an oil bath, the freshly prepared 40 ml Na_2S aqueous solution was injected into the solution, leading to the formation of a black precipitate in ~15 seconds. The mixture was heated to boiling under reflux again, held for 2 h, and allowed to cool to room temperature under Ar (BOC, 99.998%) on a Schlenk line. The products were collected by centrifuge, washed with DIW and ethanol several times, and dried in ambient atmosphere at 50 ºC for 12 h. Scaled-up syntheses were performed with eight-fold precursor concentrations, i.e. using 1.2 mol NaOH, 80 mmol $SnCl_2 \cdot 2H_2O$, and 160 mmol Na_2S; the products demonstrated phase purity and morphology identical to the products synthesised at lower precursor concentrations.

In a typical synthesis of S-substituted SnSe ($SnS_{0.1}Se_{0.9}$) nano/micro-plates, 40 ml of fresh NaHSe aqueous solution, prepared by mixing 10 mmol Se (Aldrich, 99.5%) and 20 mmol $NaBH_4$ (Alfa, 98%) in 40 ml DIW within a single-neck round-bottom flask ($2Se + 4NaBH_4 + 7H_2O \rightarrow 2NaHSe + Na_2B_4O_7 + 14H_2\uparrow$), was promptly injected into the 90 ml SnS nano/micro-plates suspension obtained after being boiled for 2 h. The new suspension was heated to boiling under reflux again, held for another 2 h (or 1 min, to study the formation mechanism of the S-substituted SnSe), and allowed to cool to room temperature under Ar on the Schlenk line. The products were collected by centrifuge, washed with DIW and ethanol several times and dried in ambient atmosphere at 50 ºC for 12 h. The concentration of NaHSe solution was varied to investigate its effect on the synthesis from 0.125 through 0.2, 0.225, 0.25, 0.2875 to 0.375 mol L^{-1} (corresponding to NaHSe:Na_2SnO_2 molar ratios of 0.5:1, 0.8:1, 0.9:1, 1:1, 1.15:1, 1.5:1, respectively). Due to the excellent controllability and repeatability of this synthesis method, scale-up syntheses of $SnS_{0.1}Se_{0.9}$ (NaHSe:Na_2SnO_2 molar

ratio of 1:1) with six-fold precursor concentrations were performed, i.e. using 900 mmol NaOH, 60 mmol $SnCl_2 \cdot 2H_2O$, 120 mmol Na_2S and 240 ml NaHSe solution (0.25 mol L^{-1}); the products demonstrated phase purity and morphology identical to the products synthesised at lower precursor concentrations. The synthesised samples used for characterisation and performance evaluation were loaded in glass vessels and stored in an Ar-filled MBraun glove box (< 0.5 ppm H_2O, < 0.5 ppm O_2) to avoid possible reaction with ambient air.

Materials Characterisation and Performance Evaluation. The phase composition and crystal structures of the as-prepared samples were investigated by powder X-ray diffraction (PXD), using a PANalytical X'pert Pro MPD diffractometer in Bragg-Brentano geometry (Cu $K\alpha_1$ radiation, λ = 1.5406 Å). Diffraction data were collected at room temperature with a step size of 0.017 ° over 10 ° ≤ 2θ ≤ 90 ° for 1 h (for phase indexing) or over 10° ≤ 2θ ≤ 100° for 4 h (for structural refinement). The crystal structures of the synthesised products were refined using the Rietveld method against PXD data using the GSAS and EXPGUI software packages,[1] with the previously published SnS and isostructural SnSe structures as a basis.[2] For the refinement, a shifted Chebyschev function (type 1 within GSAS) and a Pseudo-Voigt profile function (type 2 within GSAS) were applied to model the background and peak shape, respectively. The degree of orientation of the crystal planes, estimated by the Lotgering method,[3] was calculated to evaluate the extent of texturing in the samples. The orientation degree (F) of the (h00) planes can be calculated by $F = (P-P_0)/(1-P_0)$, where $P_0 = I_0(h00)/\Sigma I_0(hkl)$, $P = I(h00)/\Sigma I(hkl)$ and I_0 and I are the peak integral intensities of a randomly orientated sample and the measured sample, respectively.

The morphological and chemical characteristics of the synthesised products were investigated by scanning electron microscopy (SEM, Carl Zeiss Sigma, 5 and 20 kV for imaging and elemental analyses, respectively), equipped with energy-dispersive X-ray spectroscopy (EDS, Oxford Instruments X-Max 80). The synthesised powders were spread on a conductive carbon tape that was mounted on a standard SEM sample stub. Microstructure, crystal structure and chemical composition were further characterised by transmission electron microscopy (TEM) using an FEI Titan Themis

200 electron microscope equipped with an X-FEG Schottky field emission gun and a Super-X windowless EDS detector, operating at an accelerating voltage of 200 kV. TEM images, high resolution TEM (HRTEM) images, selected area electron diffraction (SAED) patterns and high angle annular dark field (HAADF) images were recorded using an FEI Ceta 16-megapixel CMOS camera. To prepare TEM samples from $SnS_{0.1}Se_{0.9}$ pellets, powders were peeled from the pellet surface using a blade followed by a gentle crushing using mortar and pestle. The peeled $SnS_{0.1}Se_{0.9}$ powders, solution-synthesised $SnS_{0.1}Se_{0.9}$ powders or solution-synthesised SnS powders were dispersed in ethanol by sonication for 30 s to obtain a uniform dispersion. Then 2-5 drops of the suspension were dropped on to a 3 mm diameter holey C-coated Cu TEM grid.

Thermogravimetric-differential thermal analysis (TG-DTA) of the samples was performed using a Netzsch STA 409 thermal analyser located in an Ar-filled MBraun glove box (< 0.1 ppm H_2O, < 0.1 ppm O_2). Approximately 25 mg from the pellets of either SnS or $SnS_{0.1}Se_{0.9}$ was heated to 700 °C in an alumina pan under flowing Ar (60 ml min^{-1}) at a heating rate of 5 ºC min^{-1}. Diffuse reflectance UV-Vis (DR-UV-Vis) spectra were measured using a Shimadzu, UV-2600 spectrophotometer (λ = 400-1300 nm) using powdered samples crushed from SnS or $SnS_{0.1}Se_{0.9}$ pellets, which were spread into a thin uniform layer on a layer of $BaSO_4$ powder. The diffuse reflectance (R) was expressed as the Kubela-Munk function, $F(R) = (1-R)^2/(2R)$, where h is Planck's constant, v is the frequency of vibration ($v = 1/\lambda$) and hv is the photon energy. The indirect optical band gap was determined from a plot of $(hvF(R))^{1/2}$ vs. hv by taking the tangent to the point of inflection and measuring the intercept on the x-axis (in eV).

The synthesised tin chalcogenide nano/micro-plates were sintered into dense pellets in order to measure their thermoelectric performance. Once dried, the synthesised SnS and $SnSe_{0.1}Se_{0.9}$ powders were used for sintering without grinding or sieving. SnS powder was loaded into a graphite die (diameter of 15 mm) within an N_2-filled glovebox and treated in a spark plasma sintering (SPS) furnace (FCT HP D 25, FCT System GmbH) at 500 °C for 5 min under vacuum (~4 Pa) with a uniaxial pressure of ~60 MPa. The SPS process resulted in a pellet with a diameter of 15 mm, a thickness of

6 mm and a density of 5.17 g cm^{-3} (corresponding to a relative density of ~99% of the theoretical value). Similarly, $SnS_{0.1}Se_{0.9}$ powder was loaded into a graphite die (diameter of 15 mm) within a N_2-filled glovebox and sintered using the SPS furnace (500 °C for 5 min at ~4 Pa with a uniaxial pressure of ~60 MPa), to yield a pellet of equivalent dimensions and a density of 5.95 g cm^{-3} (corresponding to a relative density of ~98% of the theoretical value) (denoted ***$SnS_{0.1}Se_{0.9}$-1***). $SnS_{0.1}Se_{0.9}$ pellets were also fabricated by a two-step SPS process to promote texturing:[4] $SnS_{0.1}Se_{0.9}$ powder was loaded into a graphite die (diameter of 15 mm) within the N_2-filled glovebox and then sintered using SPS at 450 °C for 5 min under vacuum (~4 Pa) with a uniaxial pressure of ~50 MPa, resulting in a pellet having a diameter of 15 mm, a thickness of ~11 mm and a relative density of ~88%; the pellet was then loaded into a graphite die having a larger diameter of 20 mm and sintered again by SPS under a uniaxial pressure of ~50 MPa into a pellet having a diameter of 20 mm, a thickness of ~5.5 mm and a relative density of ~98% of the theoretical value (denoted ***$SnS_{0.1}Se_{0.9}$-2***). The second step in the two-step SPS process can promote the superplastic flow of the unconstrained samples in the transverse direction, which can be effective in producing textured samples.[3]

The obtained SnS and $SnS_{0.1}Se_{0.9}$ pellets were cut into bars with dimensions of 12 mm x 3 mm x 2 mm, and the Seebeck coefficient (S) and electrical conductivity (σ) of the bars were measured simultaneously perpendicular to the pressing direction using a Linseis LSR-3 instrument under a helium atmosphere within a temperature range of 300-773 K. The uncertainty in the measurement of the Seebeck coefficient and electrical conductivity is 5%, leading to ~10% uncertainty for the thermoelectric power factor ($S^2\sigma$) measurement. The thermal conductivity (κ) of the $SnSe_{0.9}S_{0.1}$ pellets (***$SnS_{0.1}Se_{0.9}$-1*** and ***$SnS_{0.1}Se_{0.9}$-2***) was calculated through $\kappa = DC_p\rho$, where D, C_p and ρ are the thermal diffusivity coefficient, specific heat capacity and density, respectively. The $SnS_{0.1}Se_{0.9}$ pellets were also cut into rectangular pellets with dimensions of 5 mm x 5 mm x 2 mm, and the diffusivity, D of the $SnS_{0.1}Se_{0.9}$ pellets (Figure S25a) was measured perpendicular to the pressing direction using a Netzsch LFA 457 instrument under an Ar atmosphere within a temperature range of 300-773 K. The specific heat capacity, C_p of $SnSe_{0.9}S_{0.1}$ (Figure S25b) was calculated from the weighted average[5] of

the reported C_p values for SnSe[6] and SnS.[7] The density of the pellets was measured by the Archimedes method. The uncertainty in the κ measurement is ~8%. Electronic thermal conductivity (κ_e) was estimated using the Wiedemann-Franz law ($\kappa_e = L\sigma T$, where L is the Lorentz number; L of 1.5 x 10^{-8} V^2 K^{-2} was applied[6]), and lattice thermal conductivity (κ_L) was calculated by subtracting κ_e from κ. The ZT was calculated through $ZT = S^2\sigma T/\kappa$, with an uncertainty of ~15%. Hall effect measurements perpendicular to the SPS pressing direction were performed on a Nanometrics HL5500 Hall system using a Van der Pauw configuration. The pellets were shaped into 5 mm x 5 mm squares with a thickness of 2 mm. Silver contacts were placed at the edges of the surface of the sample with a size of 500 μm x 500 μm. The Hall coefficient (R_H) was measured directly by the Hall system. Hall carrier density (n_H) and mobility (μ_H) were calculated using $n_H = (eR_H)^{-1}$ and $\mu_H = R_H\sigma$, where e is the electron charge.

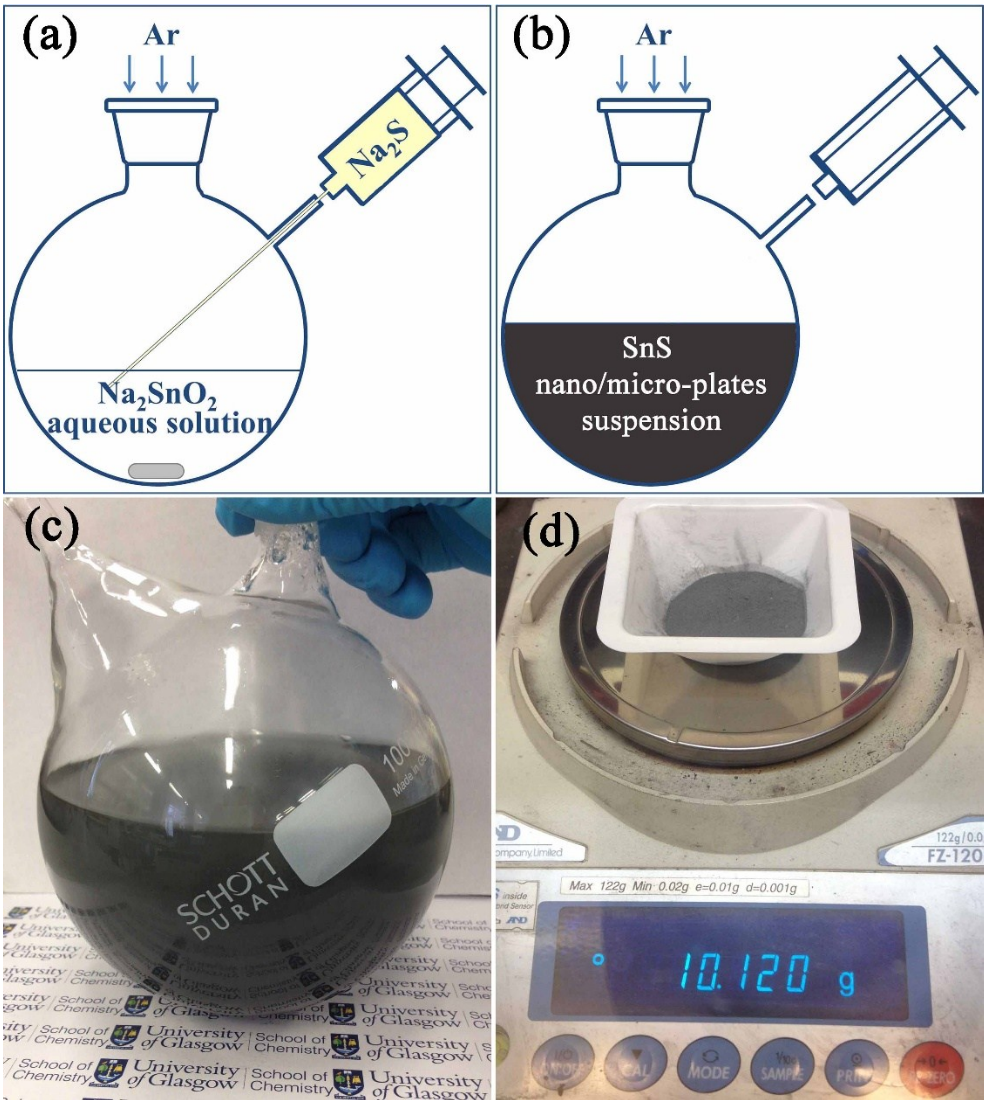

Figure S1. Schematics showing: (a) injection of $Na_2S_{(aq)}$ into $Na_2SnO_{2(aq)}$ to trigger the direct precipitation ($Na_2S + Na_2SnO_2 + 2\ H_2O \rightarrow SnS + 4\ NaOH$) and (b) formation of SnS nano/micro-plates; digital photographs showing: (c) the SnS nano/micro-plates suspension directly after synthesis and (d) a typical yield of SnS nano/micro-plates (~10.1 g) produced from a scaled-up one-pot synthesis.

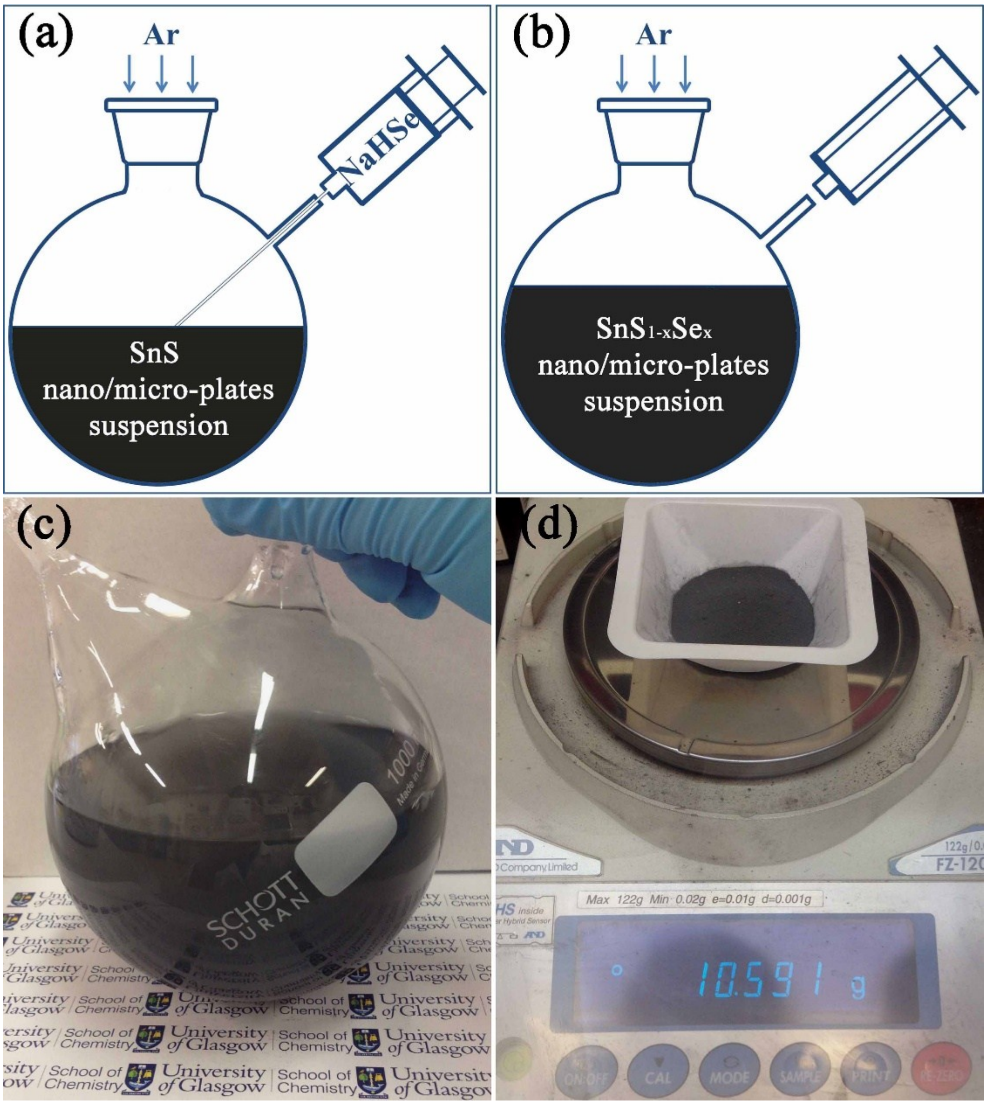

Figure S2. Schematics showing: (a) injection of $NaHSe_{(aq)}$ into an SnS aqueous suspension to trigger the anion exchange ($SnS + x\ NaHSe + x\ NaOH \rightarrow SnS_{1-x}Se_x + x\ Na_2S + x\ H_2O$; $0.5 \leq x \leq 1$) and (b) formation of $SnS_{0.1}Se_{0.9}$ nano/micro-plates; digital photographs showing: (c) the $SnS_{0.1}Se_{0.9}$ nano/micro-plates suspension directly after synthesis and (d) a typical yield of $SnS_{0.1}Se_{0.9}$ nano/micro-plates (~10.6 g) produced from an anion exchange synthesis.

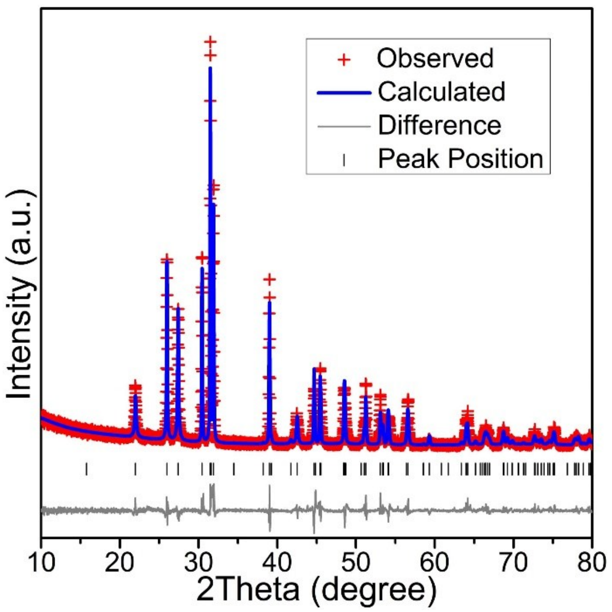

Figure S3. Profile plots from Rietveld refinement against PXD data for SnS nano/micro-plates synthesised after 2 h of boiling.

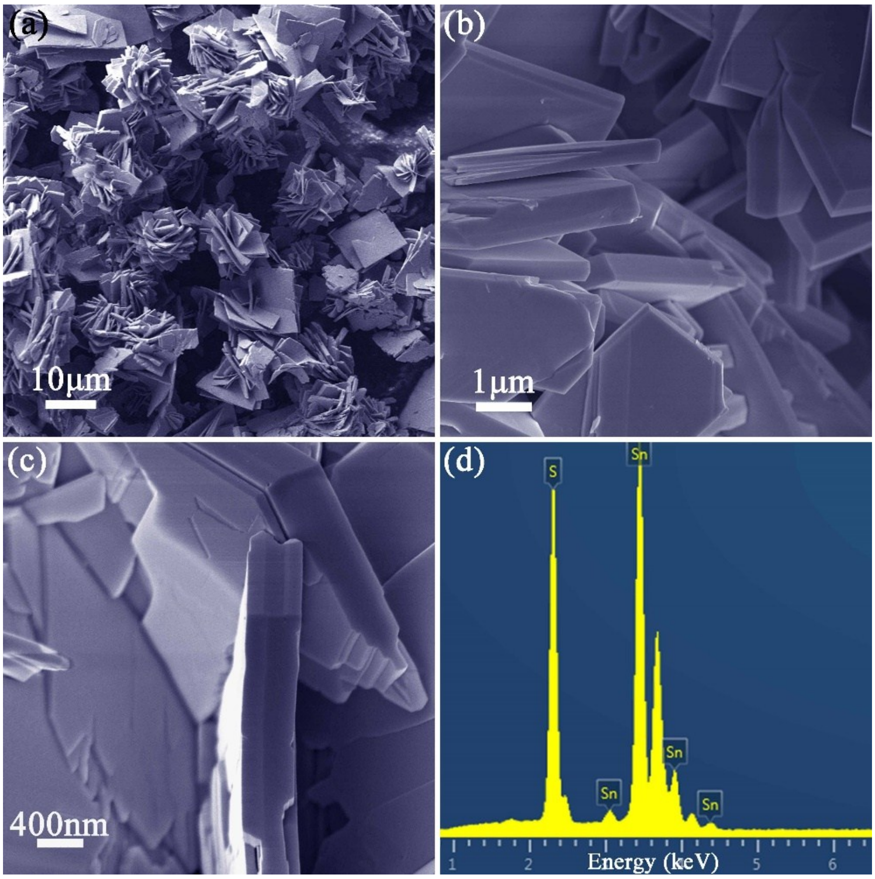

Figure S4. (a-c) SEM images and (d) EDS spectrum of SnS nano/micro-plates synthesised after 2 h of boiling.

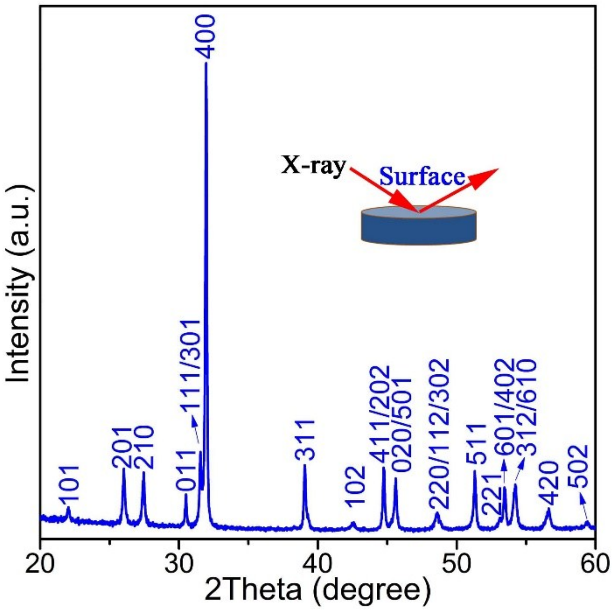

Figure S5. PXD pattern from an SnS pellet; the inset image indicates the orientation of the pellet in the X-ray beam.

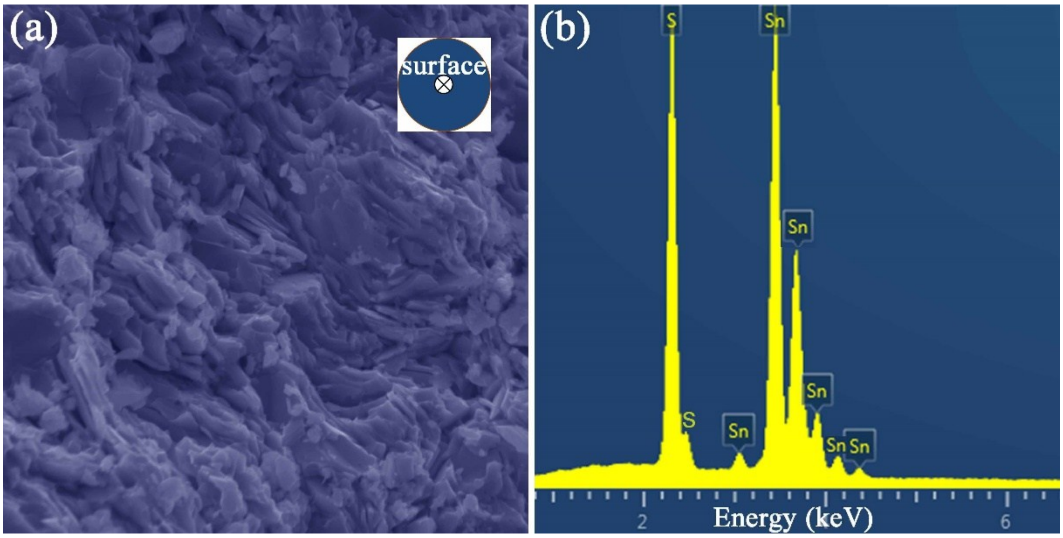

Figure S6. (a) SEM image (with viewing direction indicated in the inset image) and (b) EDS spectrum from an SnS pellet.

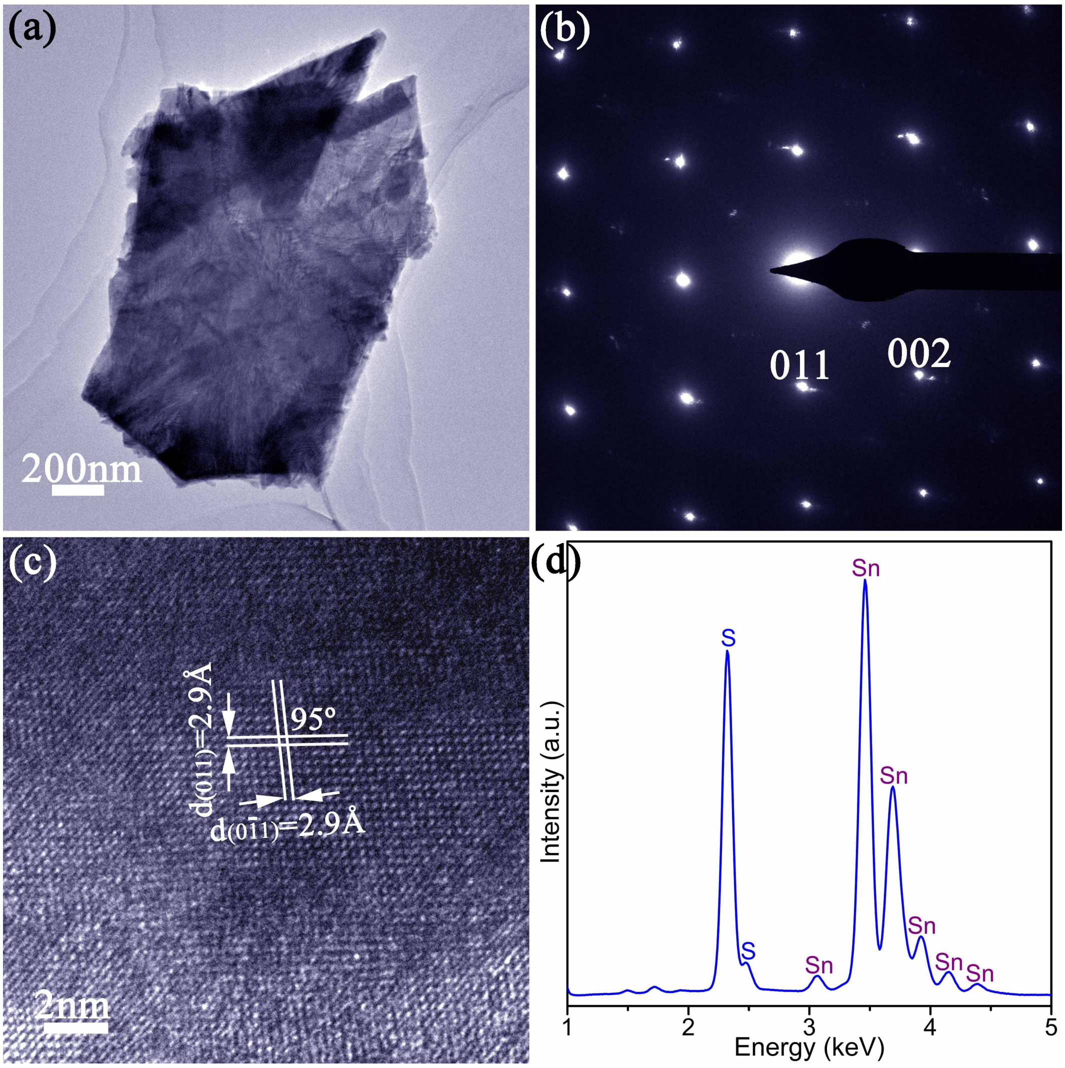

Figure S7. TEM characterisation of a SnS plate peeled from a SnS pellet: (a) TEM image; (b) SAED pattern along the [100] zone axis; (c) HRTEM image; (d) EDS spectrum.

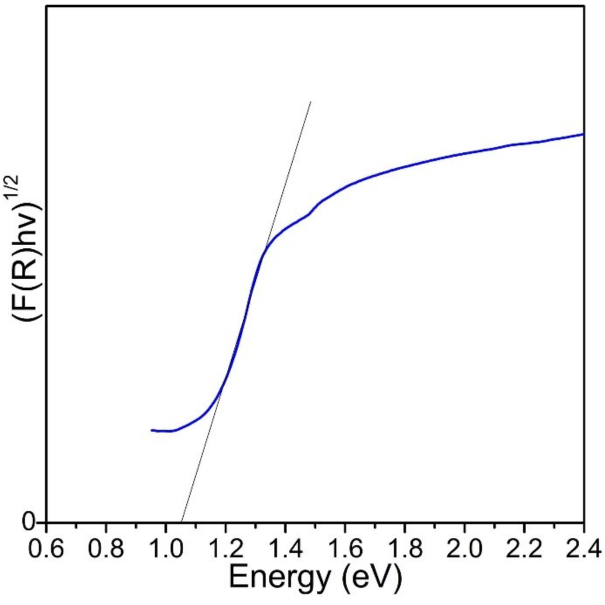

Figure S8. $[F(R)h\nu]^{1/2}$ *vs* energy plot from DR-UV-Vis spectroscopy data for an SnS pellet.

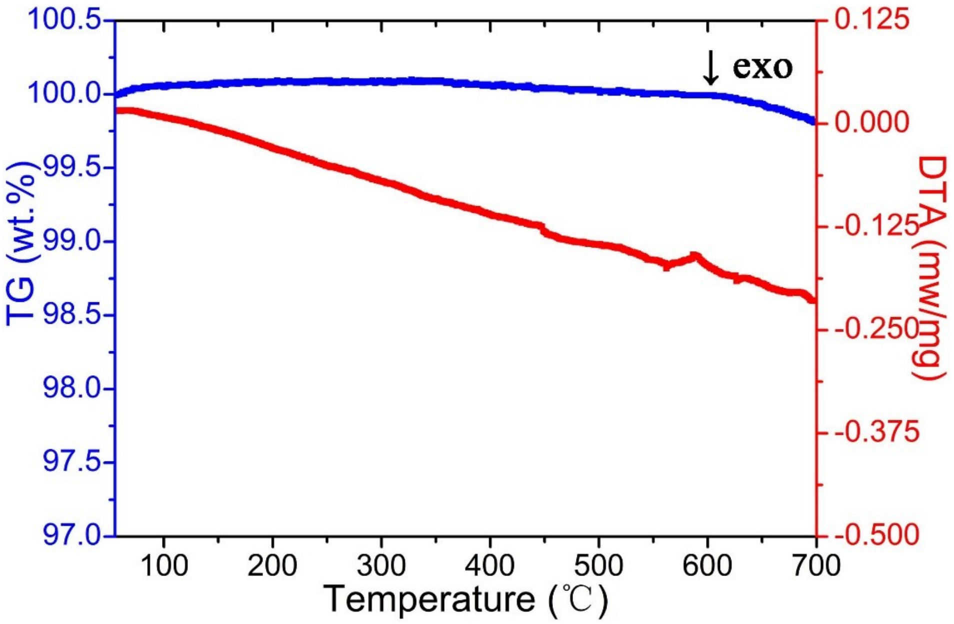

Figure S9. TGA (blue) and DTA (red) profiles from an SnS pellet heated to 700 °C under $Ar_{(g)}$.

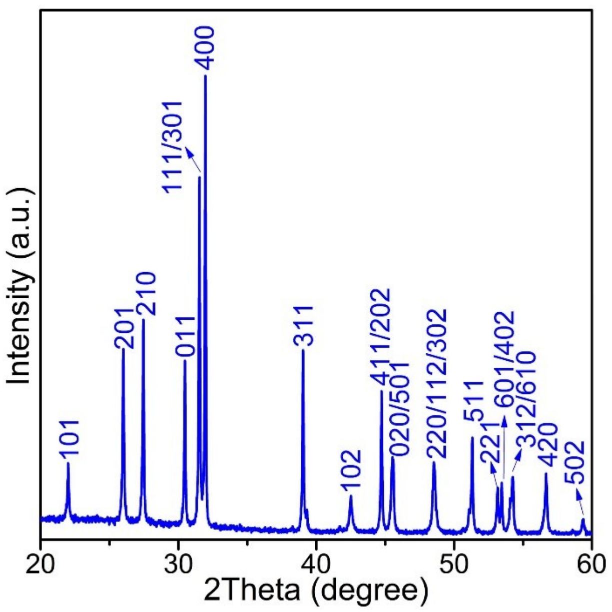

Figure S10. PXD pattern of crushed sample of SnS pellet after TG-DTA under Ar flow up to 700 ºC.

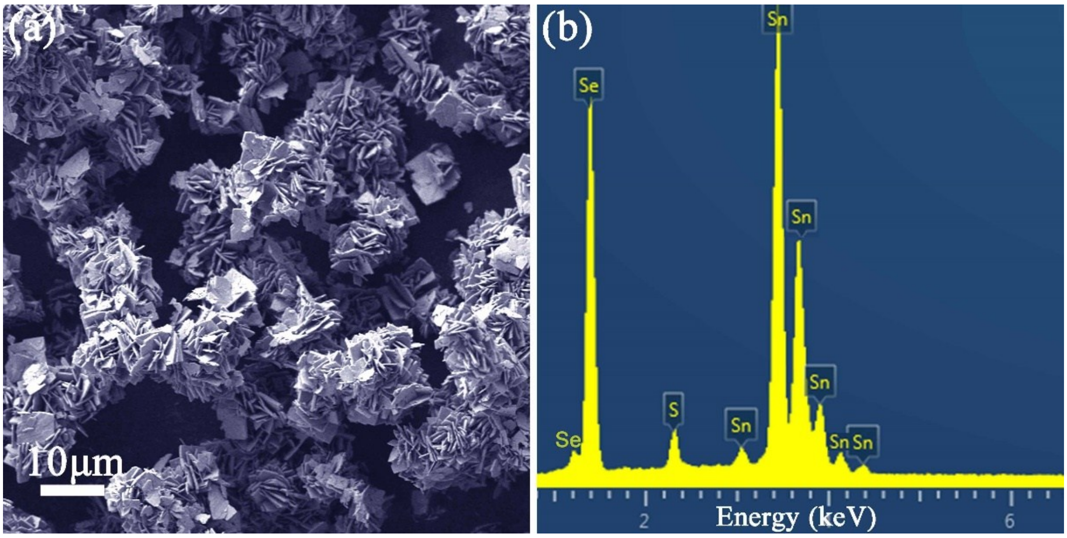

Figure S11. (a) SEM image and (b) EDS spectrum of $SnS_{0.1}Se_{0.9}$ nano/micro-plates synthesised via 2 h anion exchange from SnS nano/micro-plates ($NaHSe$:Na_2SnO_2 molar ratio is 1:1).

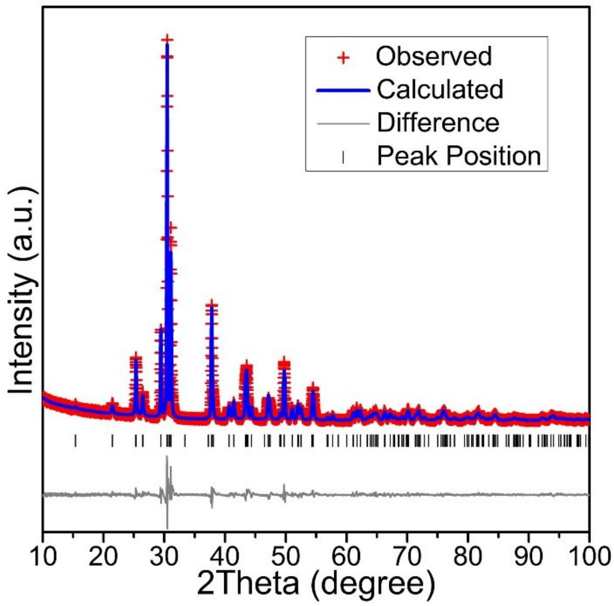

Figure S12. Profile plots from Rietveld refinement against PXD data for $SnS_{0.1}Se_{0.9}$ nano/micro-plates synthesised via 2 h anion exchange from SnS nano/micro-plates (NaHSe:Na_2SnO_2 molar ratio is 1:1).

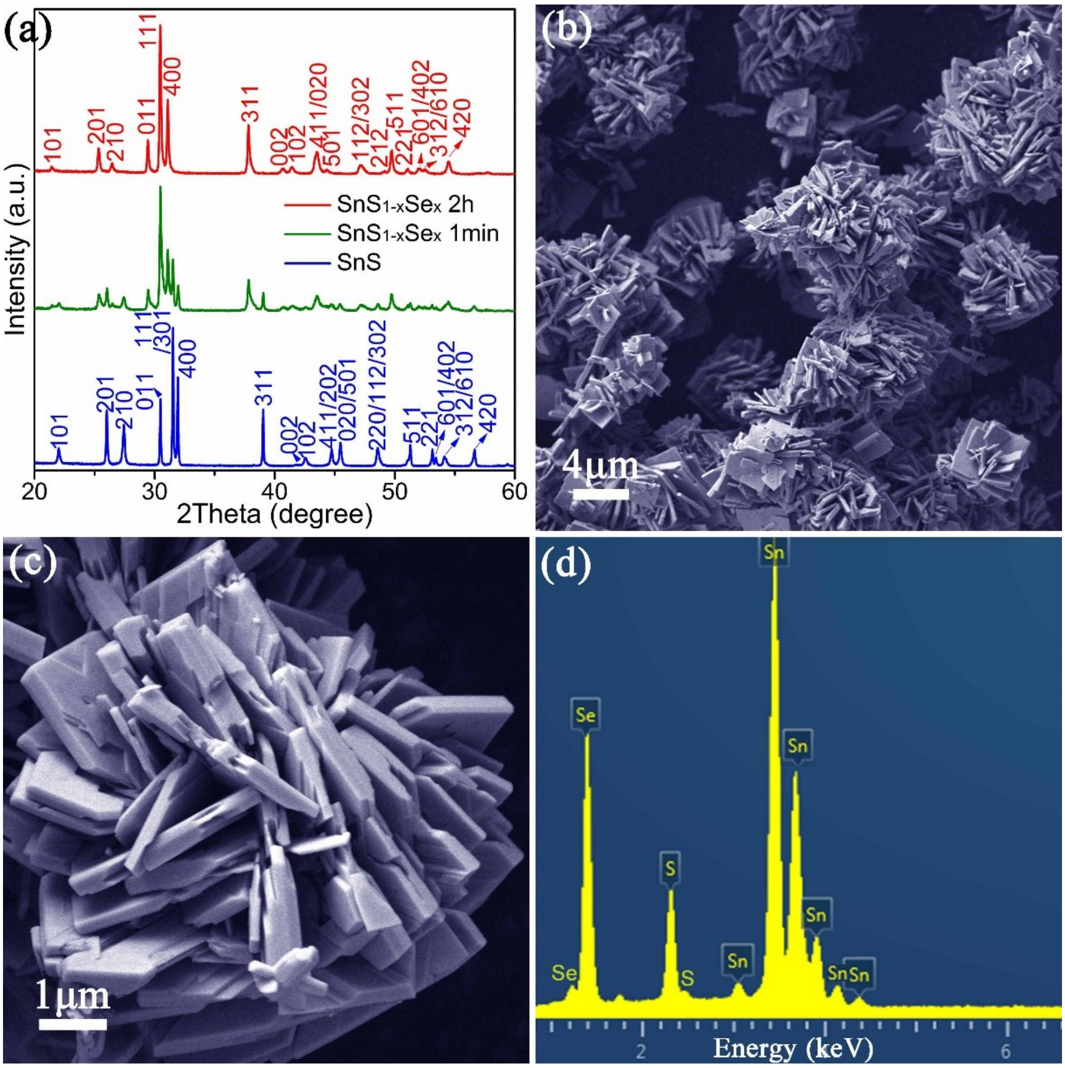

Figure S13. Characterisation of the product after boiling 1:1 NaHSe + SnS for 1 min showing: (a) PXD pattern (compared to SnS and the sample obtained from 2 h boiling; Rietveld refinement yields 77 wt% $SnS_{1-x}Se_x$, 23 wt% SnS for the 1 min sample); (b,c) SEM images and (d) EDS spectrum of the resulting nano/micro-plates.

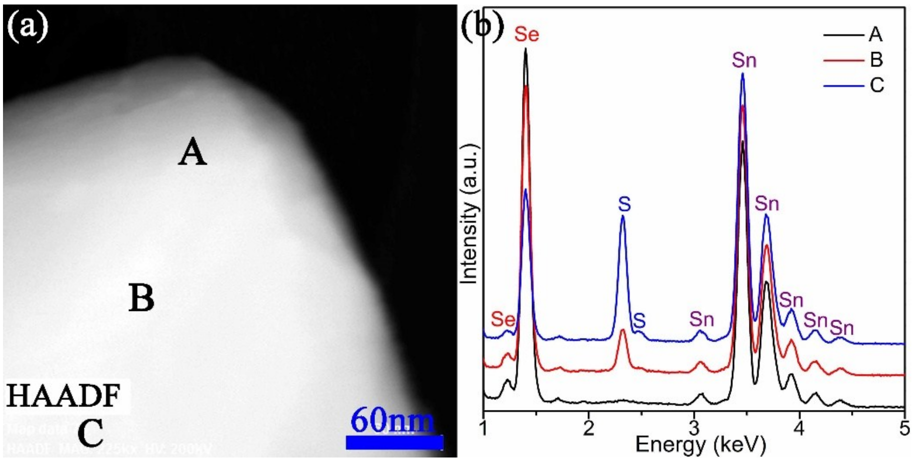

Figure S14. TEM characterisation of nano/micro-plates synthesised after boiling a 1:1 mixture of NaHSe and SnS for 1 min showing: (a) High angle annular dark field (HAADF) image, (b) EDS spectrum collected from the plate at locations A, B and C in (a).

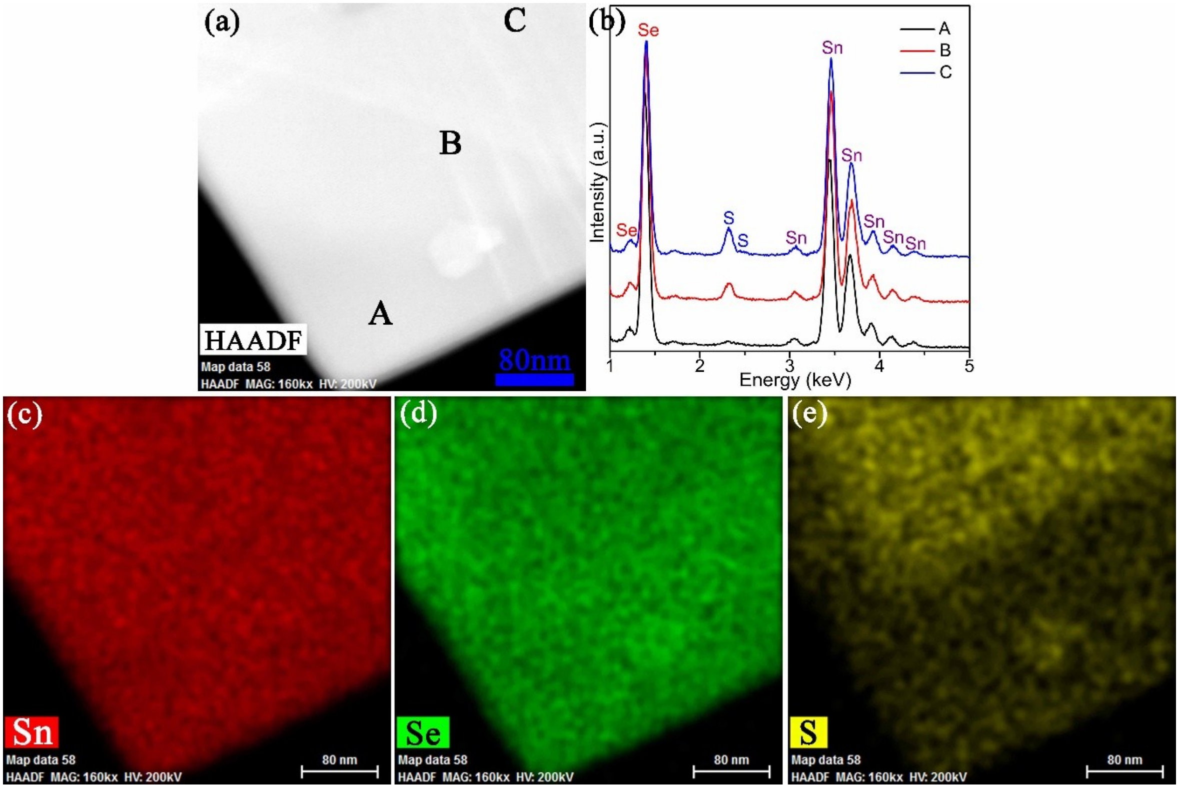

Figure S15. TEM characterisation of $SnS_{0.1}Se_{0.9}$ nano/micro-plates synthesised *via* 2 h anion exchange from SnS nano/micro-plates (1:1 molar ratio of NaHSe:Na_2SnO_2) showing: (a) High angle annular dark field (HAADF) image; (b) EDS spectrum collected from the plate locations A, B and C in (a); (c-e) elemental maps for Sn (red), Se (green) and S (yellow) respectively from the image in (a).

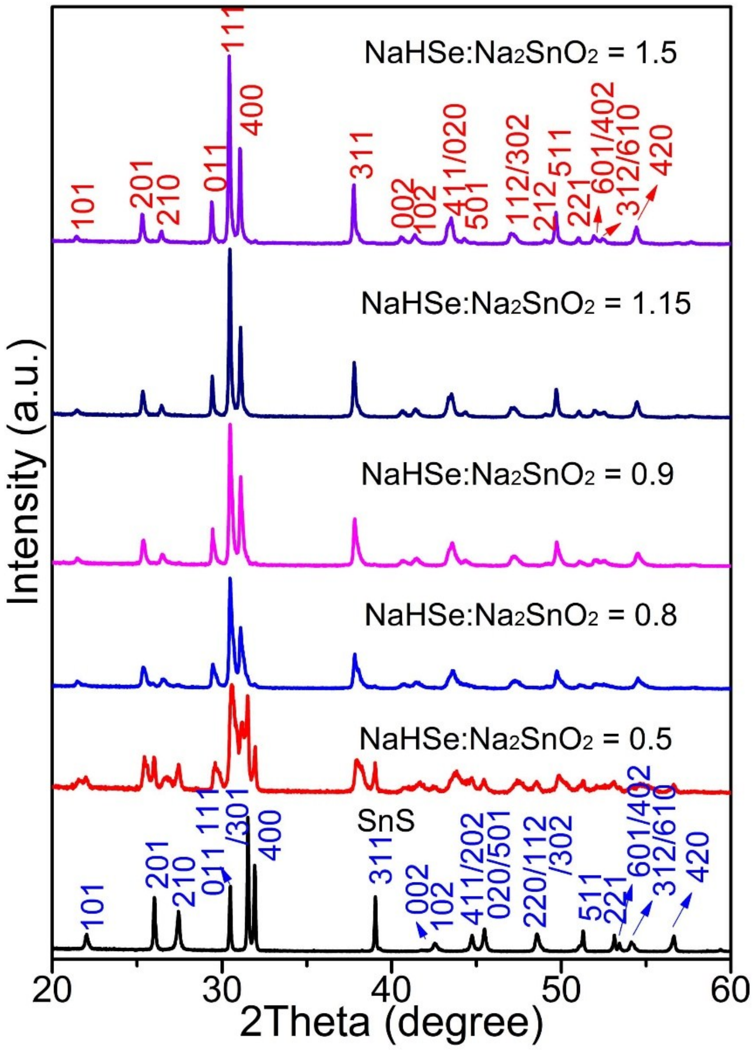

Figure S16. PXD patterns of $SnS_{1-x}Se_x$ nano/micro-plates synthesised with various NaHSe:Na_2SnO_2 molar ratios from 0.5:1 to 1.5:1 *via* 2 h anion exchange from SnS nano/micro-plates.

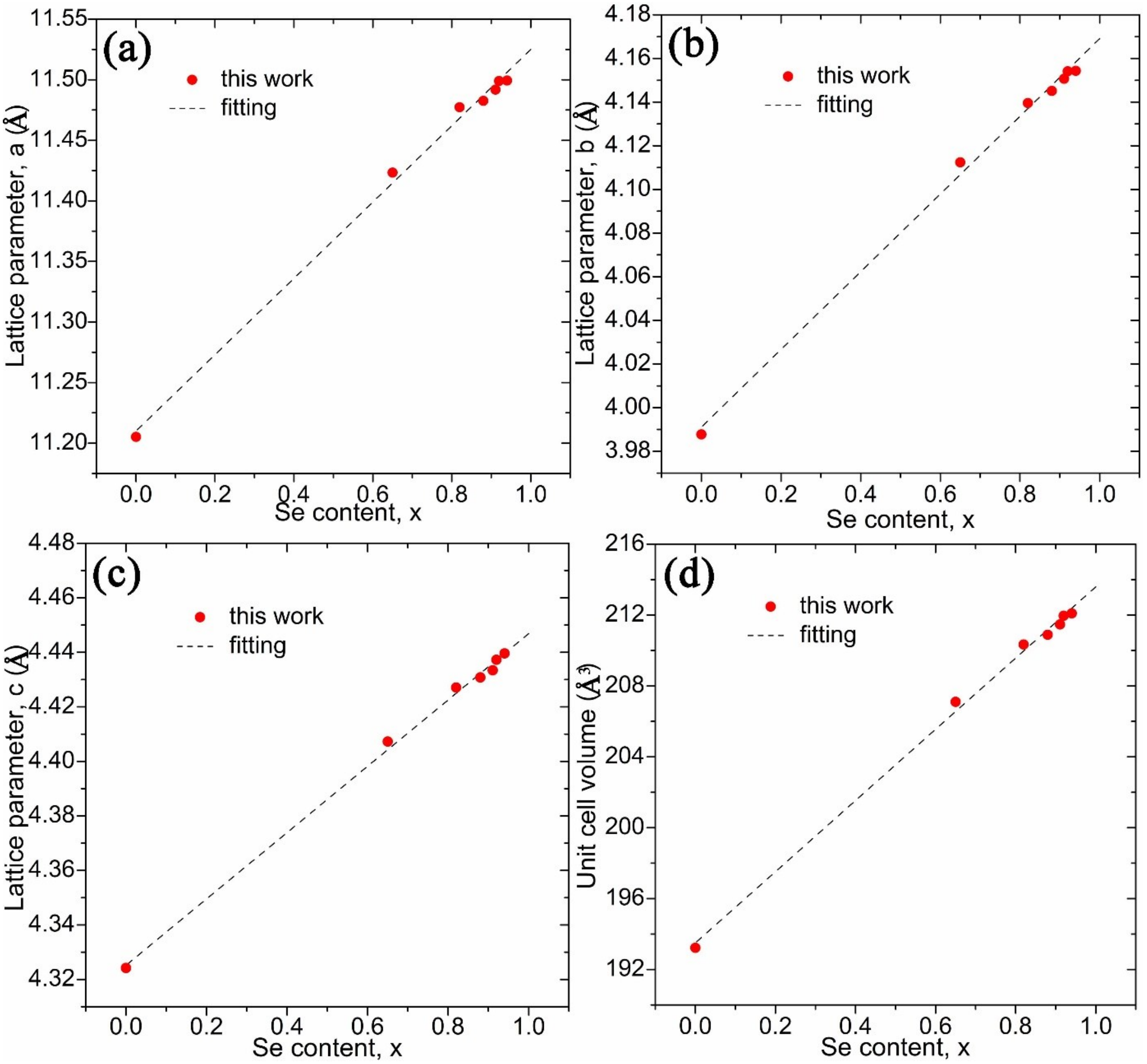

Figure S17. Lattice parameters and unit cell volumes of NaHSe:Na_2SnO_2 samples as a function of x.

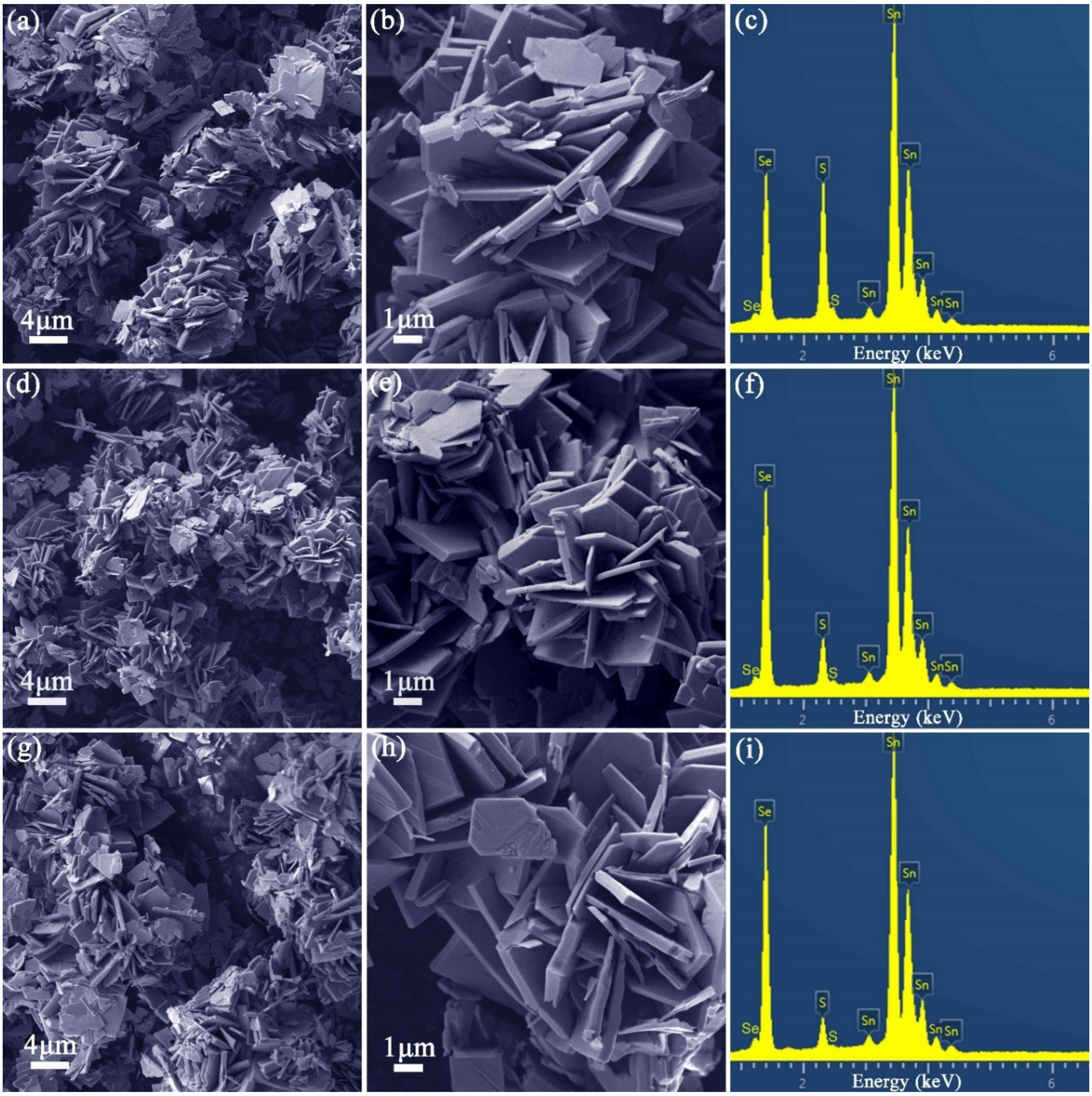

Figure S18. SEM characterisation of $SnS_{1-x}Se_x$ products synthesised with varied NaHSe:Na_2SnO_2 molar ratios *via* 2 h anion exchange from SnS nano/micro-plates showing images and EDS spectra for samples with an Se:Sn ratio of: (a-c) 0.5:1, (d-f) 0.8:1 and (g-i) 0.9:1.

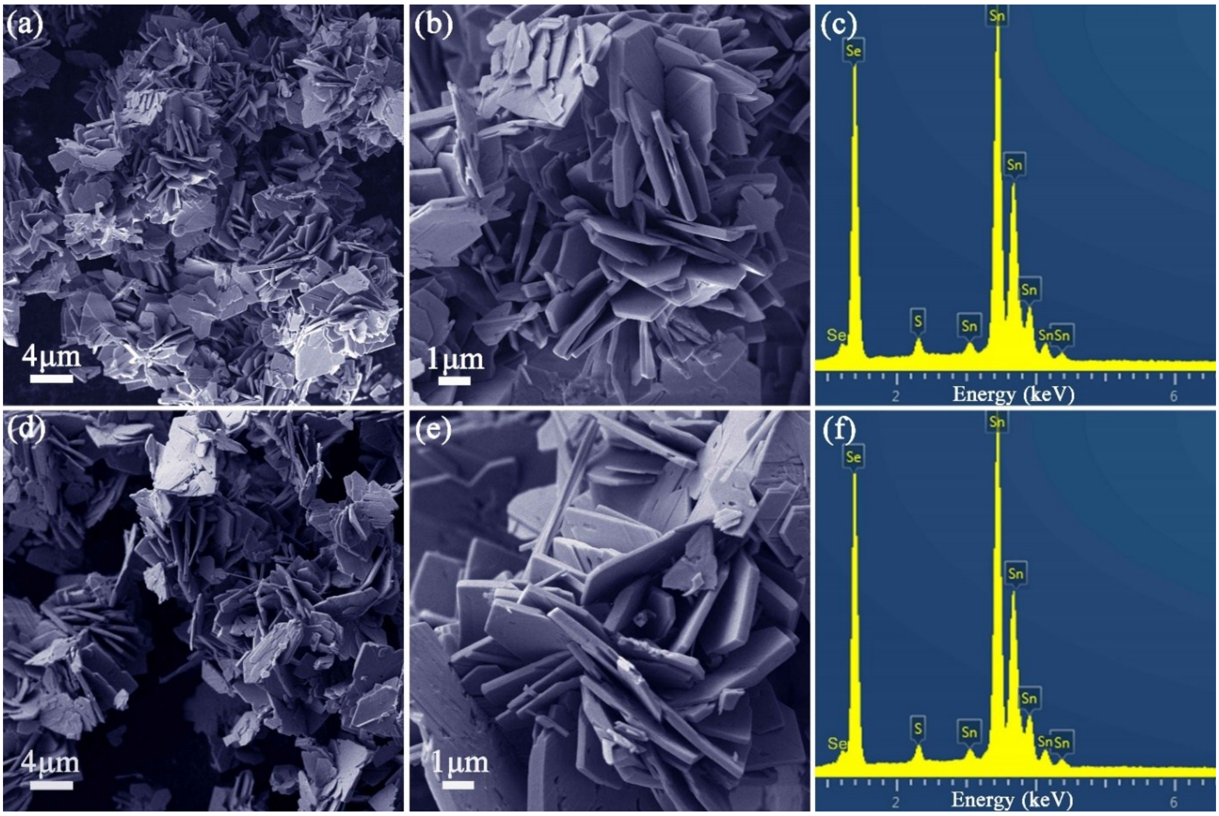

Figure S19. SEM characterisation of $SnS_{1-x}Se_x$ nano/micro-plates with NaHSe:Na_2SnO_2 molar ratios > 1:1 after 2 h anion exchange showing images and EDS spectra for samples with an Se:Sn ratio of: (a-c) 1.15:1, (d-f) 1.5:1.

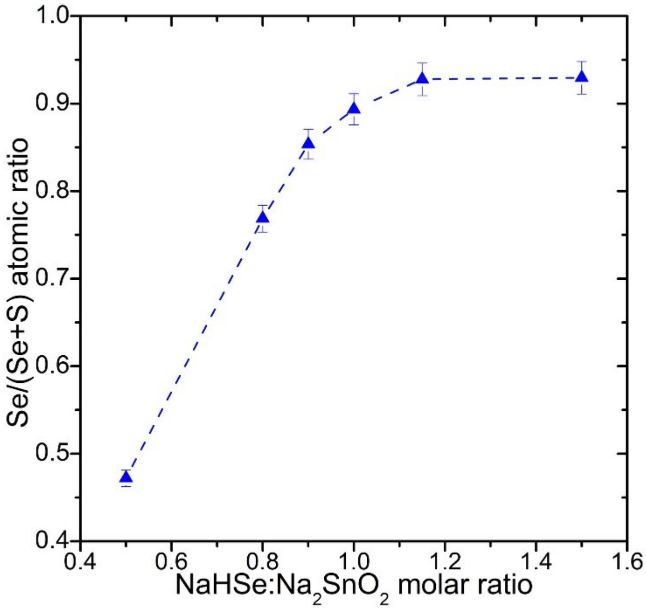

Figure S20. Plot of experimentally determined Se/(Se+S) atomic ratios for as-synthesised nano/micro-plates (from EDS) against the corresponding nominal NaHSe:Na_2SnO_2 molar ratios used in the 2 h anion exchange syntheses.

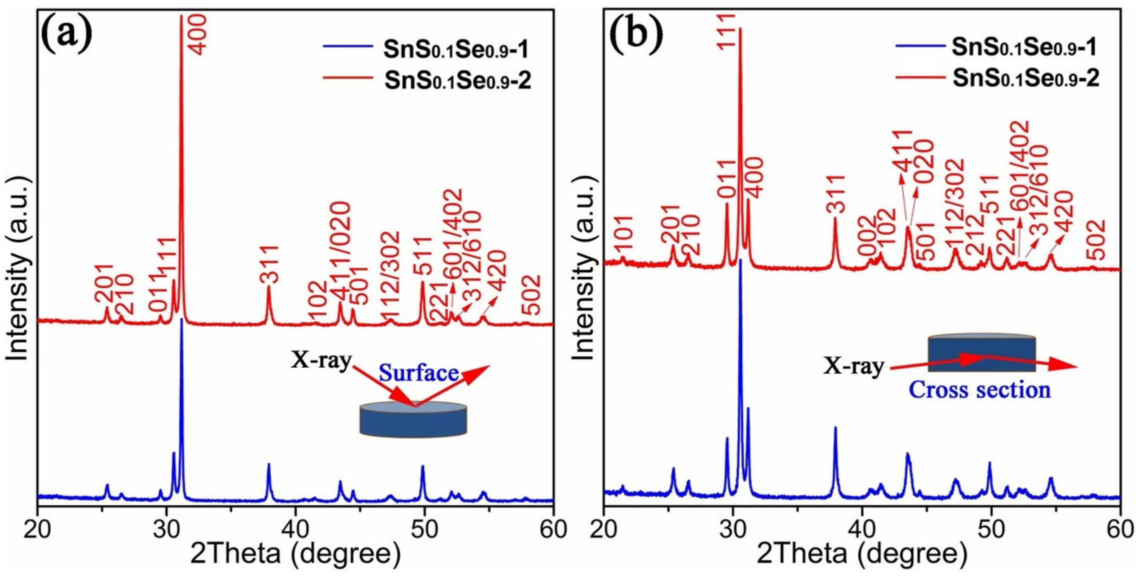

Figure S21. PXD patterns of ***$SnS_{0.1}Se_{0.9}$-1*** (blue) and ***$SnS_{0.1}Se_{0.9}$-2*** (red) collected with the pellet faces (a) perpendicular and (b) parallel to the press direction, respectively.

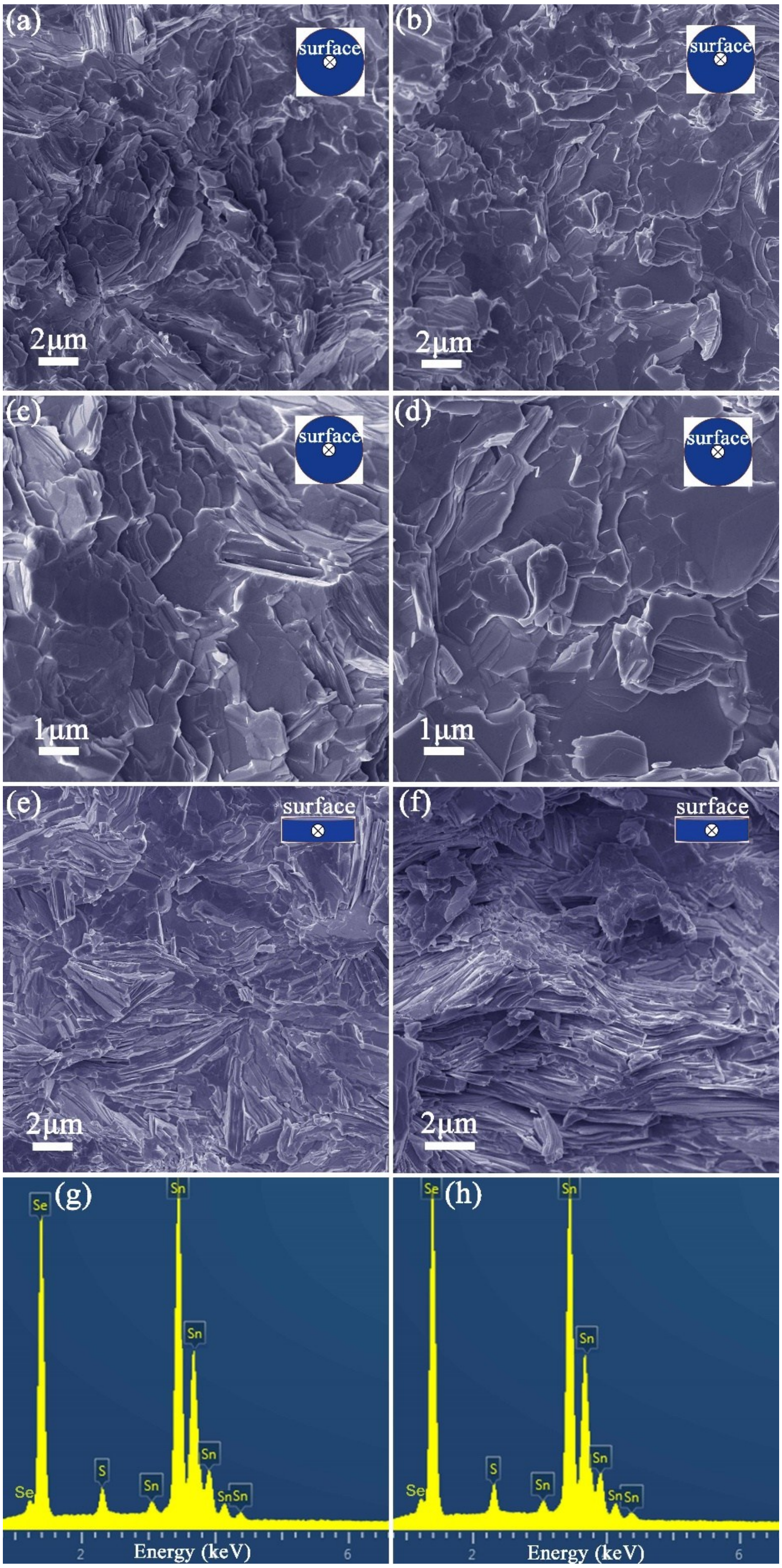

Figure S22. SEM images and EDS spectra from $SnS_{0.1}Se_{0.9}$ pellets ***$SnS_{0.1}Se_{0.9}$-1*** (a,c,e,g) and ***$SnS_{0.1}Se_{0.9}$-2*** (b,d,f,h). The SEM images show: (a-d) the cleavage surfaces (obtained by probing the sample once part of the polished face has been physically removed) and (e,f) fractured cross sections of the pellets. (The viewing direction is indicated in the inset schematics in each case.)

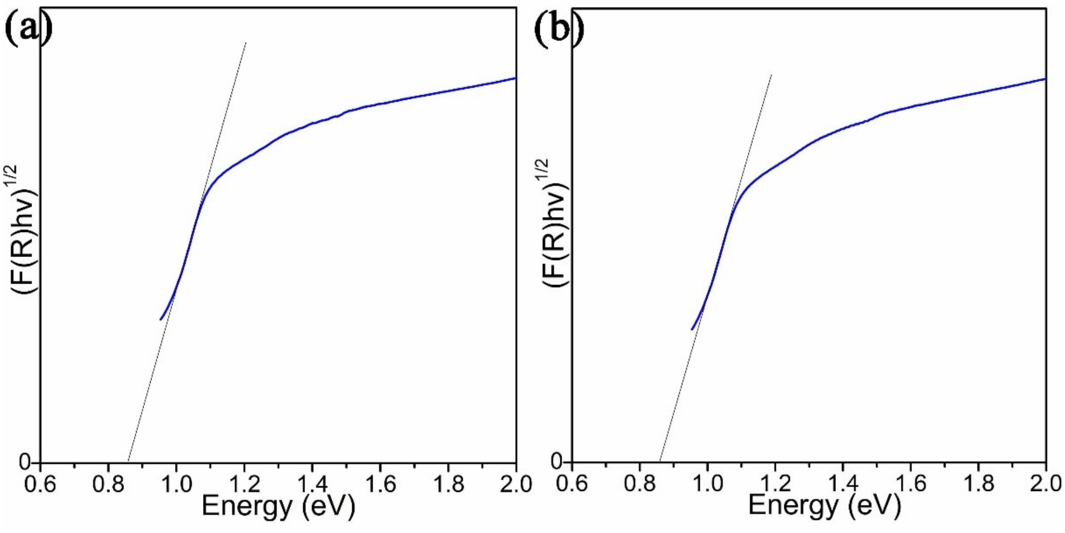

Figure S23. $[F(R)h\nu]^{1/2}$ *vs* energy (hν) plots derived from the DR-UV-Vis spectroscopy data for (a) ***$SnS_{0.1}Se_{0.9}$-1*** and (b) ***$SnS_{0.1}Se_{0.9}$-2***.

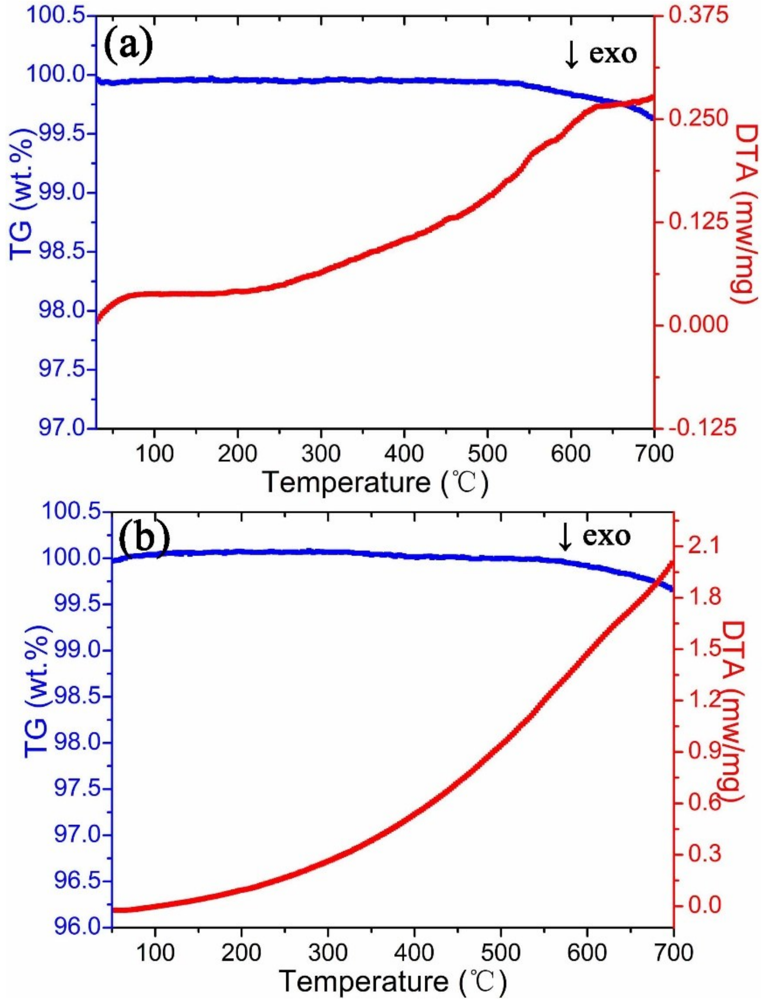

Figure S24. TGA (blue) and DTA (red) profiles of (a) ***$SnS_{0.1}Se_{0.9}$-1***, (b) ***$SnS_{0.1}Se_{0.9}$-2*** on heating to 700 °C under $Ar_{(g)}$.

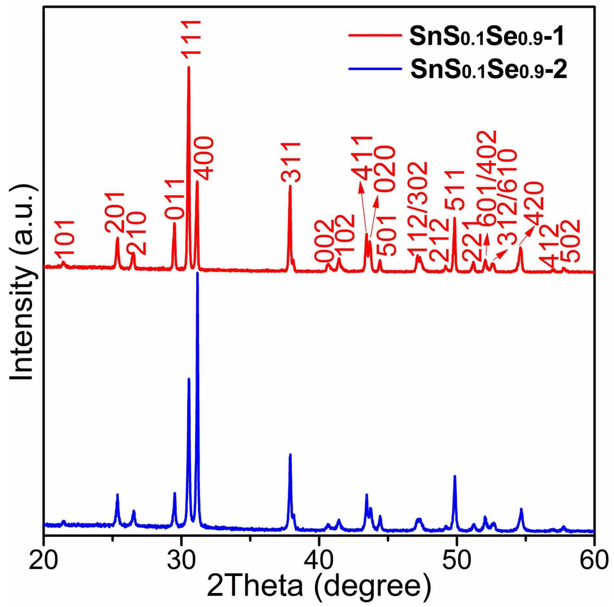

Figure S25. PXD patterns of crushed samples of ***$SnS_{0.1}Se_{0.9}$-1*** (red) and ***$SnS_{0.1}Se_{0.9}$-2*** (blue) after TG-DTA heating under flowing Ar to 700 ºC. The indices mark the reflections from the orthorhombic (space group *Pnma*) SnSe(S) structures.

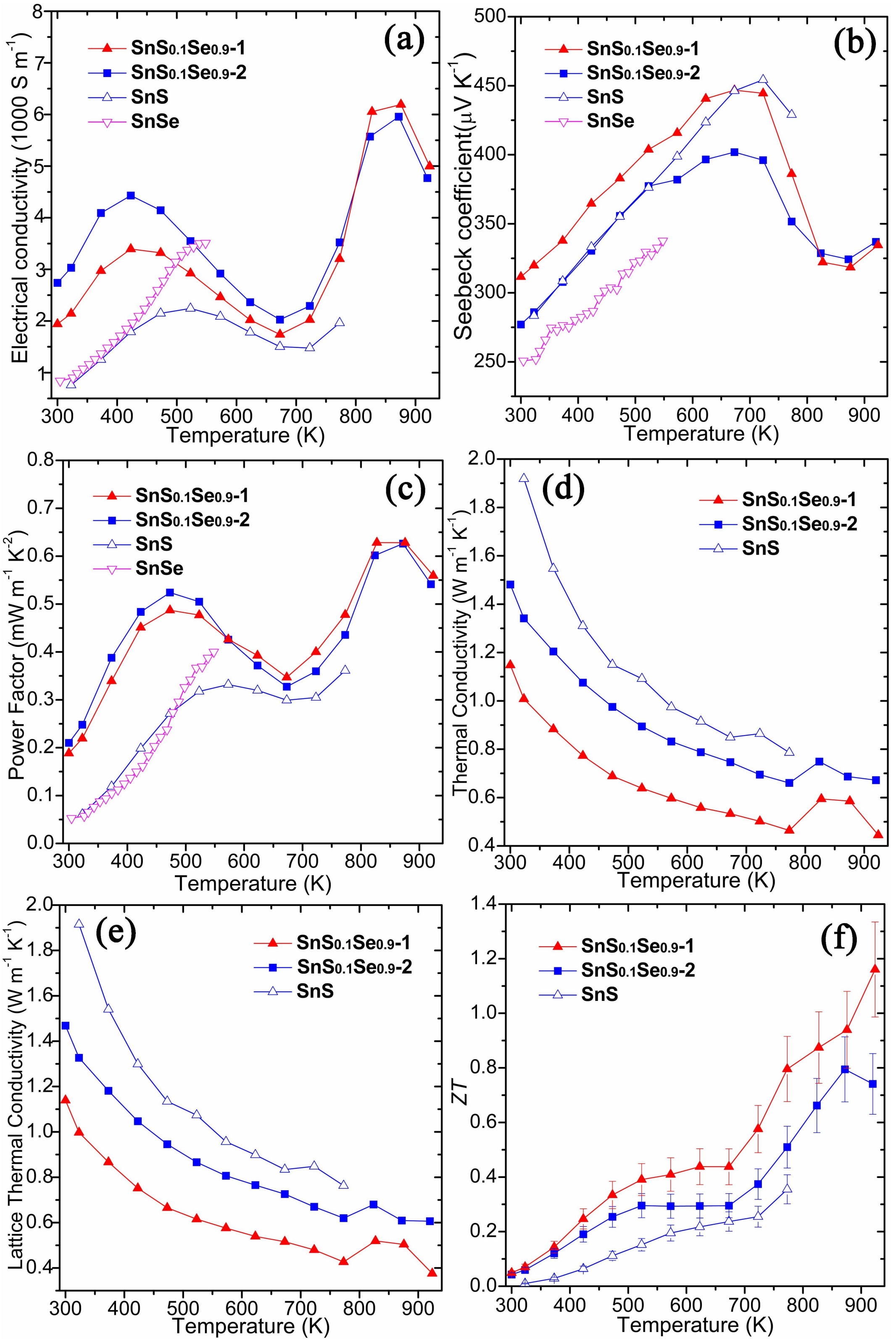

Figure S26. Thermoelectric properties of ***$SnS_{0.1}Se_{0.9}$-1***, ***$SnS_{0.1}Se_{0.9}$-2***, SnS and SnSe[8] measured perpendicular to the pressing direction: (a) the Seebeck coefficient (S), (b) the electrical conductivity (σ), (c) the power factor ($S^2\sigma$), (d) the thermal conductivity (κ), (e) the lattice κ (κ_L), and (f) ZT as a function of temperature.

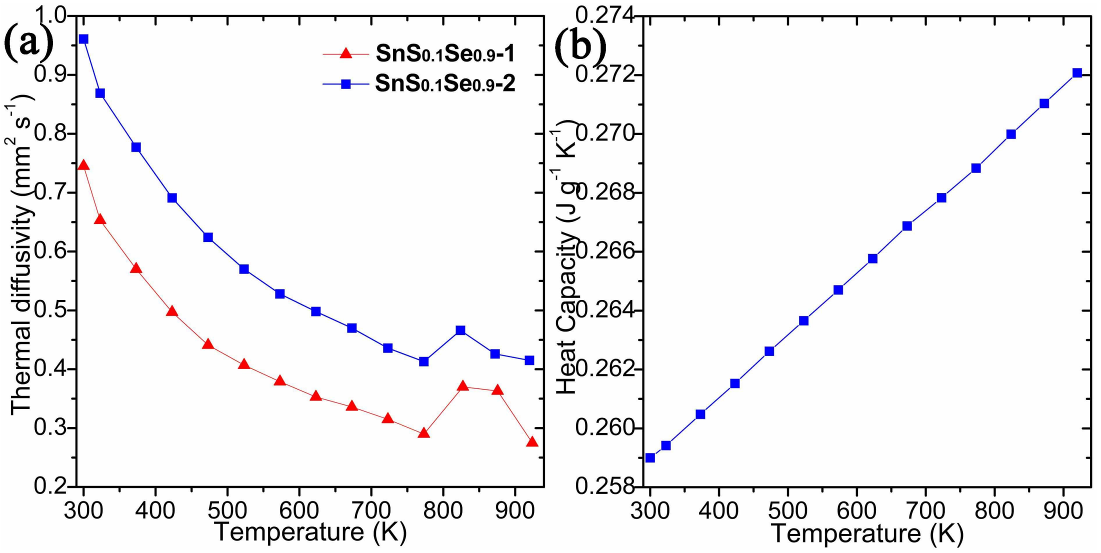

Figure S27. (a) Thermal diffusivity of ***SnS$_{0.1}$Se$_{0.9}$-1*** (red) and ***SnS$_{0.1}$Se$_{0.9}$-2*** (blue) as a function of temperature; (b) calculated heat capacity of $SnS_{0.1}Se_{0.9}$ from the weighted average[4] of the reported heat capacities, C_p, of SnSe[5] and SnS.[6]

Table S1 Crystallographic data for SnS nano/micro-plates synthesised after 2 h boiling

Chemical Formula	SnS
Crystal System	Orthorhombic
Space Group	*Pnma*
a / Å	11.2052(4)
b / Å	3.9877(2)
c / Å	4.3242(2)
Volume / Å^3	193.22(2)
Z	4
Formula Weight / g mol^{-1}	150.75
Calculated density / g cm^{-3}	5.182
R_{wp}	0.1687
R_p	0.1209
χ^2	3.291

Table S2 Atomic parameters for SnS nano/micro-plates synthesised after 2 h boiling

Atom	Wyckoff symbol	X	y	Z	100*U_{iso} (Å^2)	Occupancy
Sn	4*c*	0.1208(2)	0.2500	0.1153(4)	3.95(9)	1
S	4*c*	0.3582(7)	0.2500	0.0115(12)	2.89(27)	1

Table S3 Crystallographic data for $SnS_{1-x}Se_x$ nano/micro-plates synthesised *via* 2 h anion exchange ($NaHSe:Na_2SnO_2$ molar ratio is 1:1)

Chemical Formula	$SnS_{0.089(11)}Se_{0.911(11)}$	SnS
Crystal System	Orthorhombic	Orthorhombic
Space Group	*Pnma*	*Pnma*
a / Å	11.4919(4)	11.2075(55)
b / Å	4.1507(2)	3.9921(24)
c / Å	4.4334(2)	4.3426(27)
Volume / Å ^{3}	211.47(2)	194.29(14)
Z	4	4
Formula Weight / g mol^{-1}	193.45	150.75
Calculated density / g cm^{-3}	6.076	5.154
Phase fraction / wt.%	98.65(1)	1.35(9)
R_{wp}	0.1129	
R_p	0.0845	
χ^2	4.175	

Table S4 Atomic parameters for $SnS_{1-x}Se_x$ nano/micro-plates synthesised via 2 h anion exchange (1:1 $NaHSe:Na_2SnO_2$ molar ratio)

Atom	Wyckoff symbol	x	y	z	100*U_{iso} / Å^2	Occupancy
Sn	4*c*	0.1187(1)	0.2500	0.1010(3)	3.54(6)	1
Se	4*c*	0.3554(2)	0.2500	0.0165(4)	2.91(11)	0.92(1)
S	4*c*	0.3554(2)	0.2500	0.0165(4)	2.91(11)	0.08(1)

Table S5 Crystallographic data for $SnS_{1-x}Se_x$ nano/micro-plates synthesised with varied $NaHSe:Na_2SnO_2$ molar ratios *via* 2 h anion exchange

$NaHSe:Na_2SnO_2$ ratio	1.15		1.5	
Chemical Formula	$SnS_{0.08(2)}Se_{0.92(2)}$	SnS	$SnS_{0.06(2)}Se_{0.94(2)}$	SnS
Crystal System	Orthorhombic	Orthorhombic	Orthorhombic	Orthorhombic
Space Group	*Pnma*	*Pnma*	*Pnma*	*Pnma*
a / Å	11.4990(5)	11.1859(79)	11.4994(5)	11.1836(61)
b / Å	4.1541(2)	3.9930(34)	4.1543(2)	4.0011(24)
c / Å	4.4373(3)	4.3529(40)	4.4396(2)	4.3355(29)
Volume / $Å^3$	211.96(3)	194.42(21)	212.09(2)	194.00(15)
Z	4	4	4	4
Formula Weight / g mol^{-1}	193.84	150.75	195.02	150.75
Calculated density / g cm^{-3}	6.074	5.150	6.108	5.161
Phase fraction / wt.%	99.06(1)	0.94(11)	98.80(1)	1.20(11)
R_{wp}	0.1342		0.1431	
R_p	0.1000		0.1049	
χ^2	2.237		2.524	

Table S6 Crystallographic data for $SnS_{1-x}Se_x$ (and SnS) nano/micro-plates synthesised with varied $NaHSe:Na_2SnO_2$ molar ratios *via* 2 h anion exchange

$NaHSe:Na_2SnO_2$ ratio	0.8		0.9	
Chemical Formula	$SnS_{0.25(2)}Se_{0.75(2)}$	SnS	$SnS_{0.12(2)}Se_{0.88(2)}$	SnS
Crystal System	Orthorhombic	Orthorhombic	Orthorhombic	Orthorhombic
Space Group	*Pnma*	*Pnma*	*Pnma*	*Pnma*
a / Å	11.4773(15)	11.2209(44)	11.4826(10)	11.1937(55)
b / Å	4.1396(6)	3.9846(18)	4.1451(4)	3.9883(24)
c / Å	4.4271(8)	4.3472(20)	4.4308(5)	4.3416(27)
Volume / $Å^3$	210.34(8)	194.37(13)	210.89(5)	193.82(15)
Z	4	4	4	4
Formula Weight / g mol^{-1}	185.89	150.75	192.03	150.75
Calculated density / g cm^{-3}	5.870	5.152	6.048	5.166
Phase fraction / wt.%	94.40(7)	5.60(20)	97.86(3)	2.14(17)
R_{wp}	0.1738		0.1630	
R_p	0.1323		0.1243	
χ^2	4.069		3.863	

References:

[1] a) A. C. Larson, R. B. Von Dreele, General Structure Analysis System (GSAS); Los Alamos National Laboratory Report LAUR 86-748; Los Alamos National Laboratory, 1994; b) B. H. Toby, *J. Appl. Crystallogr.* 2001, *34*, 210-213.
[2] A. S. Avilov, R. M. Imamov, S. N. Navasardyan, *Kristallografiya* 1979, *24*, 874-875.
[3] F. K. Lotgering, *J. Inorg. Nucl. Chem.* 1959, *9*, 113-123.
[4] H. X. Yan, H. P. Ning, Y. M. Kan, P. L. Wang, M. J. Reece, *J. Am. Ceram. Soc.* 2009, *92*, 2270-2275.
[5] Y.-M. Han, J. Zhao, M. Zhou, X.-X. Jiang, H.-Q. Leng, L.-F. Li, *J. Mater. Chem. A* 2015, *3*, 4555-4559.
[6] L. D. Zhao, S. H. Lo, Y. S. Zhang, H. Sun, G. J. Tan, C. Uher, C. Wolverton, V. P. Dravid, M. G. Kanatzidis, *Nature* 2014, *508*, 373-377.
[7] Q. Tan, L. D. Zhao, J. F. Li, C. F. Wu, T. R. Wei, Z. B. Xing, M. G. Kanatzidis, *J. Mater. Chem. A* 2014, *2*, 17302-17306.
[8] G. Han, S. R. Popuri, H. F. Greer, J. W. G. Bos, W. Z. Zhou, A. R. Knox, A. Montecucco, J. Siviter, E. A. Man, M. Macauley, D. J. Paul, W. G. Li, M. C. Paul, M. Gao, T. Sweet, R. Freer, F. Azough, H. Baig, N. Sellami, T. K. Mallick, D. H. Gregory, *Angew. Chem. Int. Ed.* 2016, *55*, 6433-6437.
